# Determinant Factors and Regulatory Systems for Anthocyanin Biosynthesis in Rice Apiculi and Stigmas

**DOI:** 10.1186/s12284-021-00480-1

**Published:** 2021-04-21

**Authors:** Lingzhi Meng, Changyan Qi, Cuihong Wang, Shuai Wang, Chunlei Zhou, Yulong Ren, Zhijun Cheng, Xin Zhang, Xiuping Guo, Zhichao Zhao, Jie Wang, Qibing Lin, Shanshan Zhu, Haiyang Wang, Zhonghua Wang, Cailin Lei, Jianmin Wan

**Affiliations:** 1 Institute of Crop Sciences, Chinese Academy of Agriculture Sciences/National Key Facility for Crop Gene Resources and Genetic Improvement, Beijing, 100081 China; 2 State Key Laboratory of Crop Stress Biology for Arid Areas, College of Agronomy, Northwest A&F University, Yangling, 712100 Shaanxi China; 3 Key Laboratory of Crop Genetics and Germplasm Enhancement/Jiangsu Provincial Center of Plant Gene Engineering, Nanjing Agricultural University, Nanjing, 210095 Jiangsu China

**Keywords:** Anthocyanin, Apiculus, *Oryza sativa*, *OsC1*, *OsDFR*, *OsPa*, *OsPs*, Stigma

## Abstract

Anthocyanins cause purple, brown or red colors in various tissues of rice plants, but the specific determinant factors and regulatory systems for anthocyanin biosynthesis in almost all tissues remain largely unknown. In the present study, we mapped and isolated two complementary genes, *OsC1* encoding a R2R3-MYB transcriptional factor and *OsDFR* encoding a dihydroflavonol 4-reductase, which are responsible for the purple coloration of apiculi and stigmas in *indica* cultivar Xieqingzao by the map-based cloning strategy. We also identified two tissue-specific pigmentation genes, *OsPa* for apiculi and *OsPs* for stigmas, by phylogenetic analysis of all anthocyanin biosynthesis-associated bHLH transcriptional factors in maize and rice, CRISPR/Cas9 knockout and transcriptional expression analysis. The OsC1, OsPa and OsPs proteins are all localized in the nucleus while the OsDFR protein is localized in the nucleus and cytoplasm, and the *OsC1* and *OsDFR* genes are preferentially strongly expressed in both purple-colored tissues while the *OsPa* and *OsPs* genes are preferentially strongly expressed in apiculi and stigmas, respectively. OsC1 specifically interacts with OsPa or OsPs to activate *OsDFR* and other anthocyanin biosynthesis genes, resulting in purple-colored apiculi or stigmas. *OsC1* itself does not produce color but can produce brown apiculi when functioning together with *OsPa*. Loss of function of *OsDFR* alone leads to brown apiculi and straw-white stigmas. Genotyping and phenotyping of a panel of 176 rice accessions revealed diverse genotypic combinations of *OsC1*, *OsDFR*, *OsPa* and *OsPs* that enable accurate prediction of their apiculus and stigma pigmentation phenotypes, thus validating the general applicability of the *OsC1*-*OsDFR*-*OsPa* and *OsC1*-*OsDFR*-*OsPs* models to natural populations. Our findings disclosed the biological functions of *OsC1*, *OsPa* and *OsPs*, and shed light on the specific regulatory systems of anthocyanin biosynthesis in apiculi and stigmas, a further step in understanding the regulatory network of anthocyanin biosynthesis in rice.

**Supplementary Information:**

The online version contains supplementary material available at 10.1186/s12284-021-00480-1.

## Background

Anthocyanins are a major class of flavonoids that produce colored plant organs. They are involved not only in the pigmentation patterns but also in a wide range of biological functions, such as attraction of pollinators and seed dispersal agents, protection against UV radiation and high light intensity, and defense responses to abiotic and biotic stresses such as cold, drought tolerance and disease (Lin-Wang et al. 2010; Petroni and Tonelli 2011). Moreover, plant anthocyanins reputedly promote human health by protecting against certain cancers, cardiovascular diseases and other chronic disorders (Zhang et al. 2014; Zheng et al. 2019). Therefore, improving our understanding of the anthocyanin biosynthesis and its regulation in rice is a worthwhile objective.

Anthocyanin biosynthesis involves various structural and regulatory genes. The structural genes encode catalytic enzymes, including chalcone synthase (CHS), chalcone isomerase (CHI), flavanone 3-hydroxylase (F3H), flavonoid 3′-hydroxylase (F3’H), flavonoid 3′5’-hydroxylase (F3’5’H), dihydroflavonol 4-reductase (DFR), anthocyanidin synthase (ANS), and UDP-glucose: flavonoid 3-O-glucosyltransferase (UFGT) (Zhang et al. 2014). Three transcription factor families, viz. R2R3-MYB, basic helix-loop-helix (bHLH), and WD40 repeat protein (WDR), are important regulators of anthocyanin biosynthesis (Xu et al. 2015; Sun et al. 2018; Zheng et al. 2019). In maize, the MYB and bHLH proteins are encoded by two multi-gene families, *C1/Pl1* and *R1/B1*, respectively. A combination of the two types of proteins coordinately activate expression of a series of structural genes. The *R1/B1*genes, *R1*, *B1*, *Sn1*, *Lc1* and *Hopi1*, whose expressions are tissue-specific, determine distribution of pigments in different tissues (Petroni and Tonelli 2011; Oshima et al. 2019).

Earlier genetic studies of anthocyanin biosynthesis in rice revealed that pigmentation in various tissues was mainly controlled by three factors, *C* (*Chromogen*), *A* (*activator*) and *P* (*Purple*, distributor), where *C* and *A* were essentially color-producing genes and *P* was a tissue-specific regulator of both *C* and *A* (Nagao and Takahashi 1956, 1963; Takahashi 1957). Further genetic analyses suggested multiple additional loci that could be responsible for tissue-specific distribution and accumulation of anthocyanins and proanthocyanidins, such as *Pls* for coleoptile, *Pl* for leaf blade, *Psh* for leaf sheath, *Pin* for internode, *Pg* for glume, and *Ps* for stigma (Takahashi 1982; Reddy 1996). Anthocyanin biosynthesis-related genes in rice were initially isolated by referring to the sequences to known maize orthologs. These included the catalytic enzyme genes *OsCHS*, *OsCHI*, *OsANS* and *OsDFR*, and putative regulatory genes such as *OsC1*, *Ra1*/*OsB1*, *Rb*, *Rc*, *Ra2* and *OsB2* (Hu et al. 2000; Reddy et al. 1998, 2007; Druka et al. 2003; Sakamoto et al. 2001). *OsC1*, the homolog of maize *C1*, encodes a R2R3-MYB type transcription factor that is associated with apiculus pigmentation (Zhao et al. 2016). *Rc*, the homolog of maize *intensifier 1*, encodes a bHLH type transcription factor that determines pro-anthocyanin biosynthesis in pericarps and causes brown-colored rice grain. A combination of *Rc* and *Rd* (*OsDFR*) results in red grain color (Furukawa et al. 2006). *kala4* (i.e. *OsB2* or *S1*, Zheng et al. 2019), the homolog of maize *R/B*, encodes a bHLH type transcription factor that is necessary for black grain color. A structural change in the *kala4* promoter confers the black pericarp (Oikawa et al. 2015). Sun et al. (2018) proposed the C-S-A gene system for regulation of hull pigmentation; here *C1* (*OsC1*) and *A1* (*OsDFR*) collectively determine the color variation, whereas *S1* (*OsB2*) diversifies the pigmentation among tissues. Zheng et al. (2019) also proposed that *OsC1*, *OsDFR* and *OsRb* corresponding to the *C*, *A* and *P* genes coordinately determine anthocyanin biosynthesis in rice leaves. To sum up, even though a handful of R2R3-MYB and bHLH regulators have been identified individually, the comprehensive regulation systems controlling pigmentation in specific tissues and mechanism of anthocyanin biosynthesis in those tissues remain to be determined.

Purple apiculi and stigmas not only attract insects for pollination and animals for seed dispersal, but also serve as visible markers for varietal identification and purification (Chin et al. 2016; Zhao et al. 2016). The *C*-*A*-*P* system was initially established for anthocyanin pigmentation in purple apiculi in rice (Nagao and Takahashi 1956). In this system, *C* corresponds to *OsC1*, and *A* probably corresponds to *OsDFR* (Zhao et al. 2016; Sun et al. 2018; Zheng et al. 2019); however, the homology of the *P* gene remains to be determined, let alone its biological nature and function. Purple stigma has also been extensively investigated since the middle of the last century (Takahashi 1957, 1964), and again, the molecular mechanism underlying stigma pigmentation is largely unknown. Oka (1991) showed that pigmentation generally occurred only in plants having *C*, *A*, and *P*, and that some other tissue-specific genes might also be involved. Han et al. (2006), Chen et al. (2010) and Zhao et al. (2016) fine-mapped the purple stigma gene(s) to the same region as *OsC1* on chromosome 6, but *OsC1* alone failed to produce a purple stigma. Thus, isolation and characterization of the determinant genes for the pigmentation of apiculi and stigmas present a challenge for a comprehensive dissection of the regulatory network underlying anthocyanin biosynthesis and accumulation in rice.

In the present study we investigated the determinant factors and regulatory system of anthocyanin biosynthesis in rice apiculi and stigmas. Our aims were to determine (1) the genetic basis of anthocyanin biosynthesis and accumulation in apiculi and stigmas, and (2) how anthocyanin biosynthesis is specifically regulated in those tissues.

## Results

### Phenotypic Characterization of *indica* Cultivar (cv). Xieqingzao (XQZ)

**Fig. 1:**
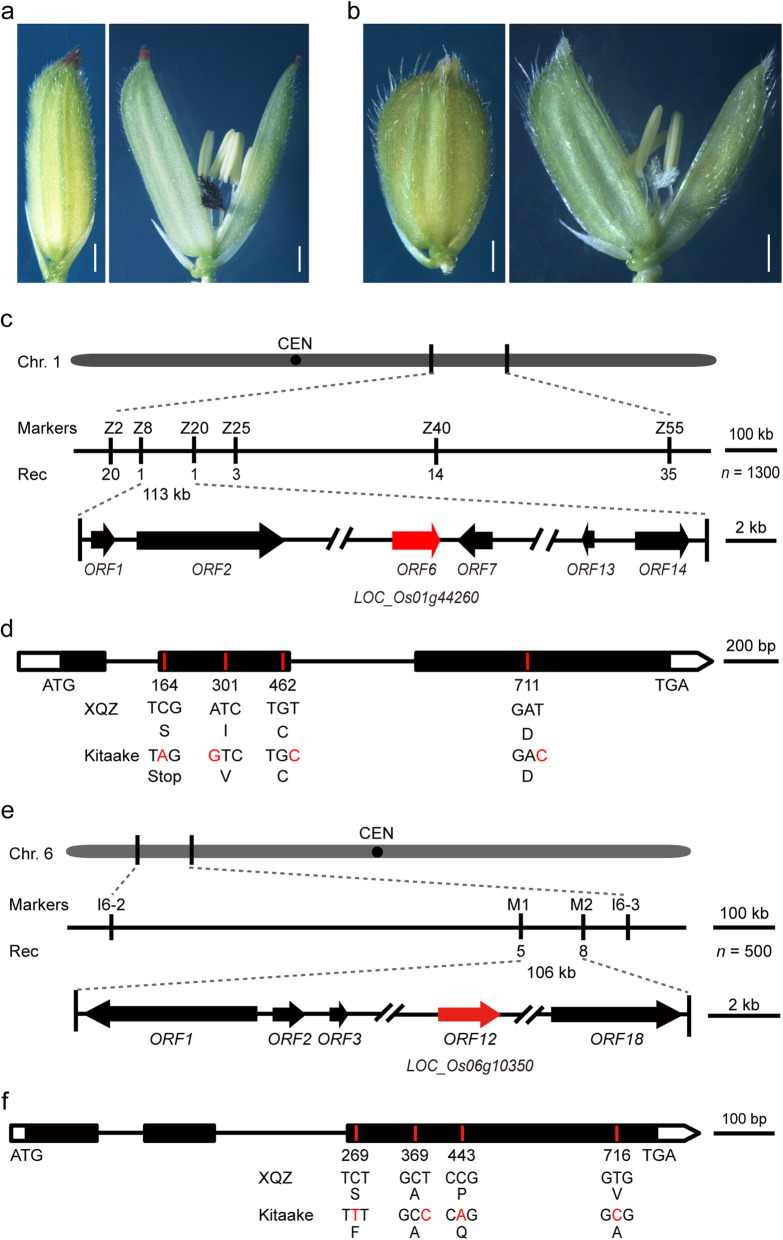
Map-based cloning of *OsC1* and *OsDFR*. **a** XQZ shows purple apiculus and stigma. Bars, 1 mm. **b** Straw-white apiculus and stigma in Kitaake. Bars, 1 mm. **c** Fine mapping of *OsDFR.* Molecular markers and numbers of recombinants are labeled above and below the filled bars, respectively. Red arrow represents target gene *OsDFR.* CEN, centromere. Chr. 1, Chromosome 1. Rec, recombinants. **d** Genomic structure and mutation sites in *ORF6* in XQZ and Kitaake. Three exons, two introns, and untranslated regions are indicated by black boxes, black lines, open boxes, respectively. Four bases substitutions in *OFR6* between XQZ and Kitaake are highlighted in red. ATG and TGA are the start and stop codons, respectively*.* **e** Fine mapping of *OsC1.* Molecular markers and numbers of recombinants are labeled above and below the filled bars, respectively. The target gene is marked in red. CEN, centromere. Chr. 6, Chromosome 6. Rec, recombinants. **f** Genomic structure and mutation sites of *OsC1* in XQZ and Kitaake. Three exons, two introns, untranslated regions are indicated by black boxes, black lines, open boxes respectively. Four bases substitutions in *ORF12* between XQZ and Kitaake are highlighted in red. ATG and TGA are the start and stop codons, respectively

Purple color in cv. XQZ was present in the apiculi and stigmas at the initial heading stage (Fig. 1a, Fig. S1a, b). The color in both tissues gradually deepened and were quite evident at anthesis (Fig. S1a, b). *Japonica* cv. Kitaake, displayed no purple color in any tissue throughout its growth cycle (Fig. 1b). The relative anthocyanin contents in the apiculi and stigmas of cv. XQZ were 6.7- and 23.4-fold higher than those in cv. Kitaake (Fig. S2), indicating that the apiculus and stigma pigmentation reflected accumulation of anthocyanins.

### Genetic Dissection of Purple Apiculi and Stigmas

F_1_ individuals derived from the cross XQZ × Kitaake exhibited purple apiculi and stigmas similar to XQZ (Fig. S3b). When the apiculus and stigma colors were compared there were three phenotypic classes: + + (apiculi and stigmas both purple), +’ - (apiculi brown, stigmas straw-white) and - - (apiculi and stigmas both straw-white) in the F_2_ population, and the numbers of plants in each fitted a 9:3:4 ratio, respectively (Fig. S3c; Table S1), indicating control by two complementary dominant genes, one of which was responsible for brown apiculi. We tentatively name the two genes as *D* and *E*, and postulated the genotypes of two parents as *DDEE* for XQZ, *ddee* for Kitaake, and *DdEe* for the F_1_. The genotypes of three F_2_ classes were postulated as *DDEE*/*DdEE*/*DDEe*/*DdEe*, *ddEE*/*ddEe*, and *DDee*/*Ddee*/*ddee*) (Fig. S3a-c).

### Mapping and Candidate Gene Analysis for Purple Apiculi and Stigmas

We firstly used 1300 F_2_ individuals with brown apiculi but straw-white stigmas (*ddEE*/*ddEe*) to map the *D* gene. The target gene was delimited to a 113 kb interval flanked by InDel (insertion/deletion) markers Z8 and Z20 on chromosome 1 L (Fig. 1c). Within this interval, the gene *LOC_Os01g44260* encodes a DFR which catalyzes the conversion of dihydroflavonols to leucoanthocyanidins, a crucial step in the biosynthesis of anthocyanins. *OsDFR* (i.e. *Rd*) was known to participate in pigmentation of pericarps, hulls and leaves (Furukawa et al. 2006; Sun et al. 2018; Zheng et al. 2019). Nucleotide sequence alignment between the *OsDFR* alleles derived from XQZ and Kitaake revealed base substitutions in the second and third exons (Fig. 1d), among which the mutations at sites 462 and 711 were synonymous, the site 301 change led to a I to V amino acid change, and the site 164 mutation (C to A) caused a premature termination of translation in Kitaake (Fig. 1d). Thus, we postulated that *OsDFR* to be a candidate for the *D* gene.

We similarly mapped the *E* gene using a total of 500 F_2_ individuals with straw-white apiculi and stigmas (*DDee*/*Ddee*/*ddee*). The target gene was delimited to a 105 kb interval flanked by InDel markers M1 and M2 on chromosome 6S (Fig. 1e). The chromogen gene *OsC1* (*LOC_Os06g10350*) located within this interval encodes a R2R3-MYB transcription factor (Fig. S7a). Subsequent DNA sequencing revealed four bases substitutions (C to T; T to C; C to A; T to C) in the third exon of *ORF12* between XQZ and Kitaake. The mutations at sites 269, 443 and 716 caused amino acid changes (S to F; P to Q; V to A) (Fig. 1f). We postulated that *OsC1* was a candidate for the *E* gene.

### Functional Validation of *OsC1* and *OsDFR*

**Fig. 2:**
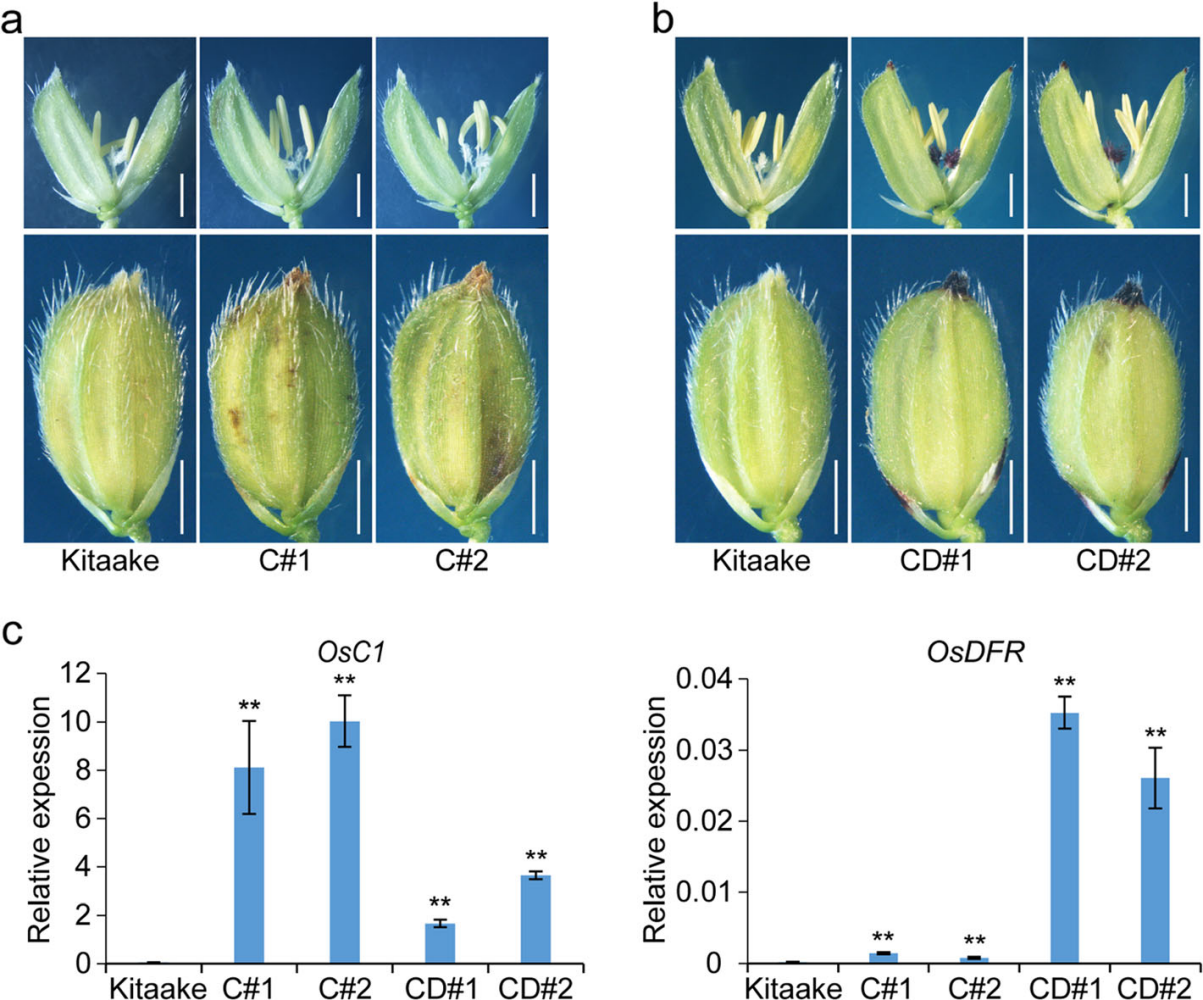
Functional validation and expression analysis of *OsC1* and *OsDFR.* **a** The straw-white apiculi became brown whereas stigmas remained straw-white after *OsC1* was introduced into Kitaake. The top, spikelets 1 day post heading; the bottom, spikelets 14 days post heading. Bars, 2 mm. **b** Both apiculus and stigma became purple when both *OsC1* and *OsDFR* were transformed into Kitaake. The top, spikelets 1 day post heading; the bottom, spikelets 14 days post heading. Bars, 2 mm. **c** Expression levels of *OsC1* and *OsDFR* in Kitaake and complementation plants. C#1, C#2, two independent *OsC1*-complemented lines. CD#1, CD#2, two independent complemented lines with *OsC1* and *OsDFR*. Data are means ± SD of three biological replicates (Student’s *t*-test: **P < 0.01)

We transformed a 4.8 kb genomic fragment containing the entire *OsC1* allele from XQZ under control of its native promoter into *japonica* cv. Kitaake with straw-white apiculus and stigma by *Agrobacterium*-mediated transformation. All positive transformants produced brown apiculi but straw-white stigmas although the pigment appeared much later than in XQZ (Fig. 2a, Fig. S4a, b, d). We then transformed a 5.9 kb genomic fragment of the entire *OsDFR* allele from XQZ under control of its native promoter into the above *OsC1*-transgenic plants. All positive digenic transformants exhibited both purple apiculi and purple stigmas with similar coloring and timing to that in XQZ (Fig. 2b, Fig. S4a, c, d). However, when the same genomic fragment of the functional *OsDFR* alone was transformed into Kitaake no positive transformant exhibited colored stigmas and apiculi. These results confirmed that complementary genes, *OsC1* and *OsDFR*, were responsible for both purple apiculi and purple stigmas, whereas *OsC1* alone gave brown apiculi only in the transgenic plants. Quantitative real-time PCR (qRT-PCR) revealed that *OsDFR* expression was significantly up-regulated with increased *OsC1* expression in both single gene (*OsC1*) and di-gene (*OsC1* and *OsDFR*) transformants relative to that in Kitaake (Fig. 2c), indicating that *OsC1* could activate *OsDFR* expression. Thus, we deduced that *OsC1* is crucial for producing color, and *OsDFR* plays a role in the *OsC1*-dependent pathway for purple coloration in rice apiculi and stigmas.

### Identification of Tissue-Specific Genes

Here, we isolated the color-producing gene *OsC1* and the activator gene *DFR*, which act coordinately to regulate the purple colors of apiculi and stigmas. Whether some tissue-specific genes were also needed for purple apiculi and stigmas remained to be determined. In maize, the bHLH-type *R1/B1* genes were demonstrated to be tissue-specific and determine the tissue distribution of pigments (Petroni and Tonelli 2011; Oshima et al. 2019). Considering the high synteny of *R/B* genes in rice and maize (Hu et al. 1996), we performed a phylogenetic analysis of all rice bHLH transcriptional factors (TFs) and all known maize bHLH TFs associated with anthocyanin biosynthesis to identify candidate tissue-specific genes. Nine rice bHLH-type TFs (Fig. S5) were found to be closest to the maize *R* genes. Among them, *Rc*, *S1* and *OsRb* had been reported to act as tissue-specific genes participating in coloration of pericarps, hulls and leaf blades, respectively (Furukawa et al. 2006; Sun et al. 2018; Zheng et al. 2019), and the other six TFs had unknown functions. We tentatively named these six TFs *HLH1* to *HLH6* (Table S2).

**Fig. 3:**
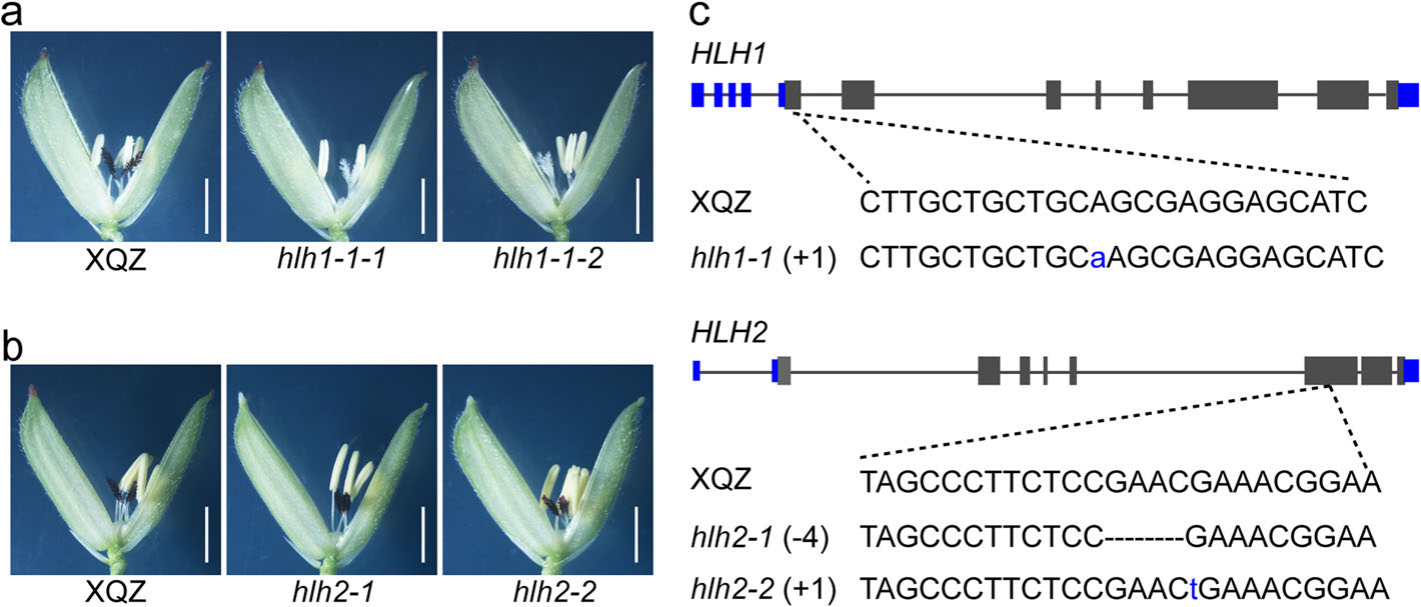
Phenotypes of *HLH1-* and *HLH2-*knockout lines in XQZ background. **a** Knockout of *HLH1* caused loss of purple stigma in XQZ. Bar, 2 mm **b** Apiculus color changed from purple to straw-white in *HLH2* knockout lines of XQZ. Bars, 2 mm. **c** Sequence alignment between XQZ and *HLH1-* and *HLH2*-knockout lines. + and - signs indicate base insertions (in blue) and deletion (by dashes), respectively, relative to XQZ. Gray boxes denote coding sequences of *HLH1* and *HLH2*, and blue boxes are the untranslated regions. *hlh1–1*, *HLH1-*transgenic knockout line. *hlh1–1-1*and *hlh1–1-2*, two individuals of *hlh1–1*-transgenic line. *hlh2–1* and *hlh2–2*, independent *HLH2-*transgenic knockout lines

To determine biological functions of the six TF-encoding genes, we knocked out all of them in the backgrounds of XQZ and the *japonica* landrace Lijiangxintuanheigu (LTH) using the CRISPR/Cas9 method. LTH possesses purple apiculi, purple stigmas (Fig. S6a, b). Only the *HLH1*- and *HLH2*-knockout mutants, i.e. *hlh1–1*, *hlh2–1* and *hlh2–2* in the XQZ background, and *hlh1–2*, *hlh1–3*, *hlh2–3* and *hlh2–4* in LTH background, displayed mutant color phenotypes (Fig. 3a-c, Fig. S6a-c). Knockouts of the other four TFs (Table S2) caused no obvious color variation in XQZ and LTH plants. The *hlh1–1* mutant had a 1 bp insertion at the site 57, and the *hlh1–2* and *hlh1–3* mutants had 4 or 6 bp deletions in the fifth extron of *LOC_Os04g47080* (*HLH1*), respectively, all of which caused loss of *HLH1* function (Fig. 3c, Fig. S6c), resulting in straw-white stigmas but purple apiculi (Fig. 3a, Fig. S6a). The *hlh2–1* and *hlh2–2* mutants contained a 4 bp and 1 bp insertions at sites 663 and 667, and the *hlh2–3* and *hlh2–4* mutants had 2 bp and 1 bp insertions in the third extron of *LOC_Os04g47040* (*HLH2*), respectively, all of which caused premature termination of translation (Fig. 3c, Fig. S6c), resulting in straw-white apiculi but purple stigmas (Fig. 3b, Fig. S6b)*.* We concluded that *HLH1* and *HLH2* are tissues-specific genes responsible for anthocyanin biosynthesis and accumulation in stigmas and apiculi of XQZ, respectively. We tentatively named them as *OsPs* (purple stigma) and *OsPa* (purple apiculus).

*OsPa* and *OsPs* both encode bHLH-type TFs containing a basic region in the N-terminal related to binding of *cis*-regulatory DNA elements and a hydrophobic HLH region in the C-terminal that functions as a homo−/hetero-dimerization domain (Fig. S7b, c). Interestingly, these two genes and another tissue-specific gene *S1* (i.e. *OsB2*), which determines anthocyanin accumulation in rice hulls, were located in a 65 kb cluster on chromosome 4 (Fig. S7d), sharing 43.81 to 57.07% identity in full-length amino acid sequence and highly conserved basic regions (73.51 to 84.24% identity) and HLH domains (84.00 to 88.00% identity) (Fig. S8; Table S3).

### Expression Patterns of *OsC1*, *OsPa*, *OsPs* and *OsDFR*

**Fig. 4:**
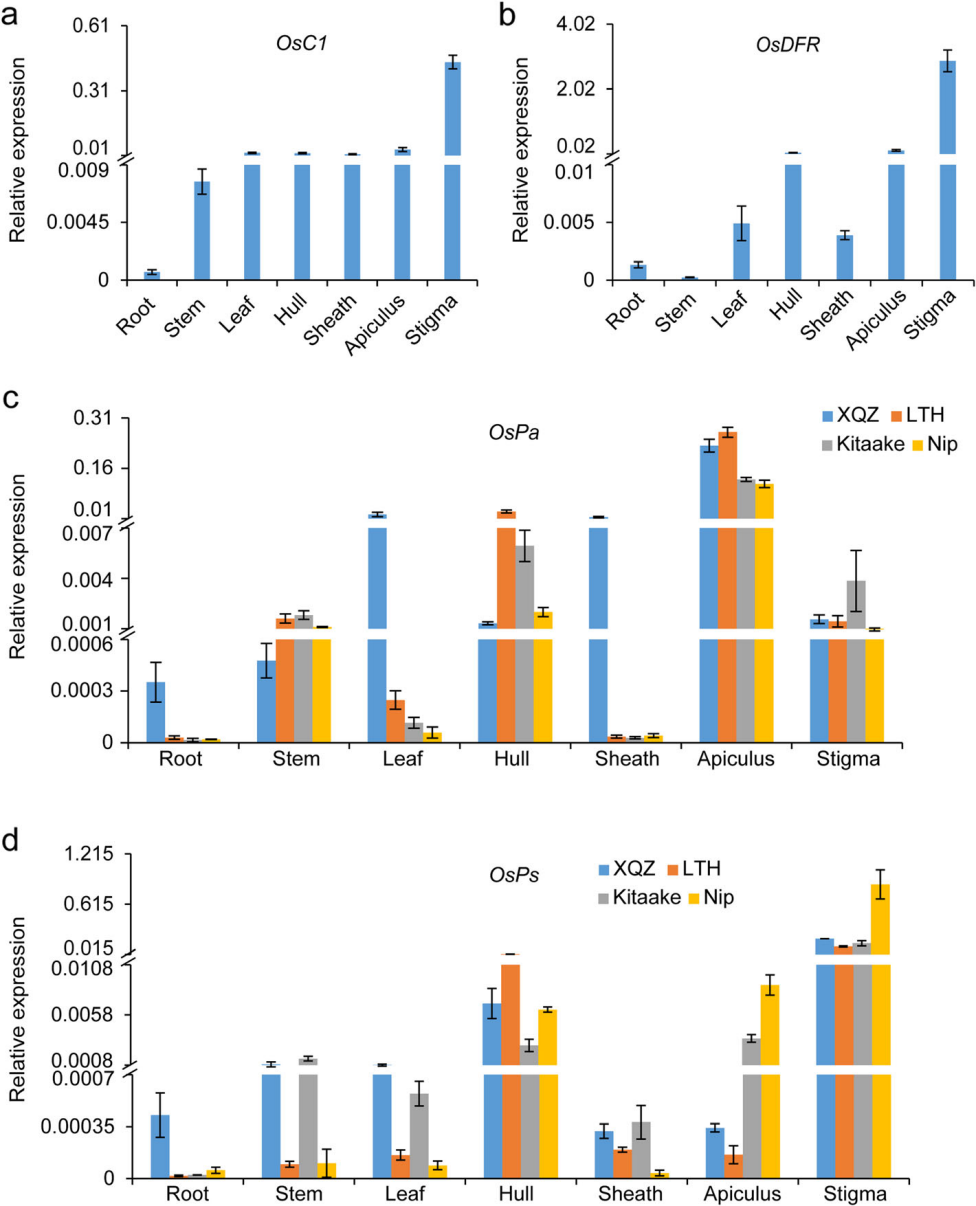
Expression patterns of *OsC1*, *OsDFR*, *OsPa* and *OsPs*. Expression analysis of *OsC1*(**a**), *OsDFR* (**b**) in XQZ. Expression patterns of *OsPa* (**c**) and *OsPs* (**d**) in XQZ, LTH, Kitaake and Nipponbare (Nip). The samples were collected from roots of 10-day-old seedlings, stems, flag leaves, hulls, leaf sheaths, apiculi, and stigmas at heading, respectively. Data are presented as means ± SD (*n* = 3)

qRT-PCR analyses revealed that *OsC1*, *OsPa*, *OsPs*, and *OsDFR* were all expressed in a range of tissues, including seedling roots, stems, leaf blades, leaf sheathes, hulls, apiculi and stigmas in XQZ plants (Fig. 4a-d), indicating that all these four genes were constitutively expressed at different developmental stages and in all tissues. However, the strongest expression of *OsC1* and *OsDFR* was detected in the respective purple-colored tissues, i.e. stigmas and apiculi, whereas there was relatively weak expression in other tissues (Fig. 4a, b). Strikingly, *OsPa* was the most strongly expressed in apiculi with expression levels 9- and 144-fold higher than that in leaves (the 2nd strongest expression tissue) and stigmas, respectively (Fig. 4c); and *OsPs* was most strongly expressed in stigmas with expression levels 28- and 588-fold higher than that in hulls (the 2nd strongest expression tissue) and apiculi (Fig. 4d). *OsPa* and *OsPs* showed similar expressional patterns in both the pigmented LTH and the non-pigmented Kitaake and Nipponbare (Fig. 4c, d). These data indicated that the preferential expression of these genes in different tissues may underlie their tissue-specific functionality.

### Subcellular Localization of OsC1, OsPa, OsPs and OsDFR

To determine the subcellular localizations of OsC1, OsPa, OsPs and OsDFR, their full-length coding sequences (CDSs) were amplified and fused to the N-terminus of green fluorescent protein (GFP) in a PAN580 vector. When transiently expressed in rice protoplasts, the OsC1-, OsPa-, OsPs-GFP signals were co-localized with the nuclear marker D53-mCherry, indicating that these three proteins were localized in the nucleus (Fig. S9), and matched their functioning in the nucleus. The OsDFR-GFP signal was co-localized with the nucleus and cytoplasm marker mCherry, indicating that the OsDFR protein is localized in the nucleus and cytoplasm (Fig. S9).

### Interactions between OsC1 and OsPa or OsPs

**Fig. 5:**
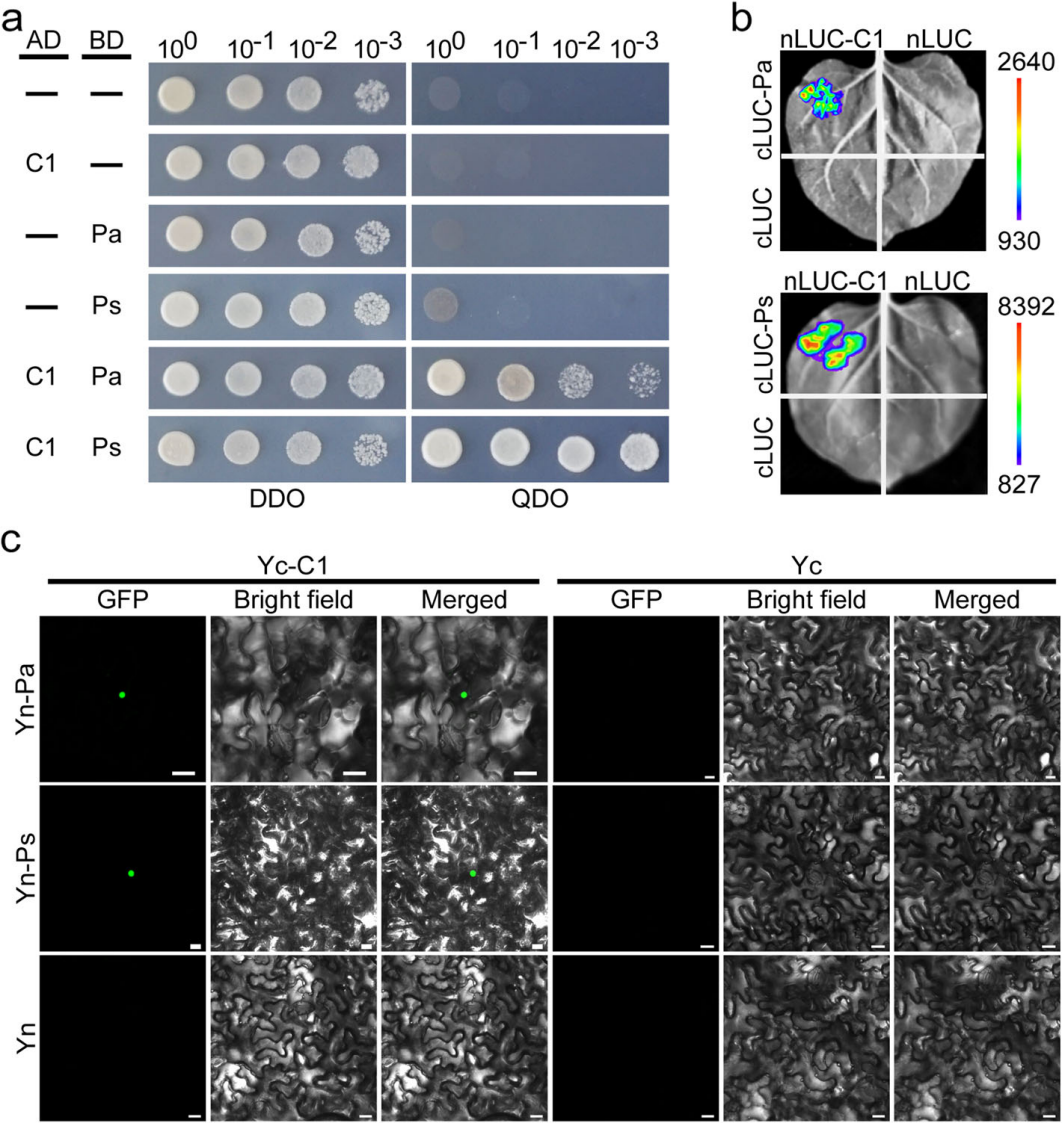
OsC1 physically interacted with OsPa and OsPs. **a** Validation of interactions of OsC1 with OsPa and OsPs in yeast two-hybrid assays after 3 days. Transformed cells were spotted on the control (DDO, SD/−Leu/−Trp) and selective (QDO, SD/−Leu/−Trp/−His/−Ade) media. **b** LCI assays certified that OsC1 interacted with OsPa or OsPs. **c** BiFC assays for the determining interaction of OsC1 with OsPa or OsPs. The full-length OsC1 protein was fused with C-terminal YFP (Yc). Full-length OsPa and OsPs proteins were fused with N-terminal YFP (Yn), respectively

Previous studies indicated MYB-type TFs interacted with tissue-specific genes to regulate expression of structural genes causing anthocyanin biosynthesis (Goff et al. 1990, 1992; Sun et al. 2018). We thus firstly used yeast two-hybrid assays to test the interactions of OsC1 with OsPa and OsPs. As expected, OsC1 indeed interacted with OsPa or OsPs (Fig. 5a). To further verify these interactions, we performed luciferase complementation imaging (LCI) assays and the bimolecular fluorescence complementation (BiFC) assays in *Nicotiana benthamiana* (*N. benthamiana*) leaves. For these assays, OsC1 was fused to N-terminal of LUC (n-LUC) to produce nLUC-C1, and OsPa and OsPs were fused to cLUC to generate cLUC-Pa and cLUC-Ps. As shown in Fig. 5b, OsC1 interacted strongly with OsPa or OsPs, displaying strong luminescence signals whereas the negative controls lacked luminescence signals. In the BiFC assays, strong fluorescence signals appeared in the cell nuclei when OsC1 was transiently co-expressed with OsPa or OsPs, but no signals appeared in cells co-expressing Yn and Yc-OsC1, Yc and Yn-Pa, Yc and Yn-Ps, or Yn and Yc as controls (Fig. 5c). These results confirmed that OsC1 could interact with OsPa or OsPs not only in yeast but also in *planta*.

### The OsC1-OsPa and OsC1-OsPs Complexes Activate Structural Gene Expression

**Fig. 6:**
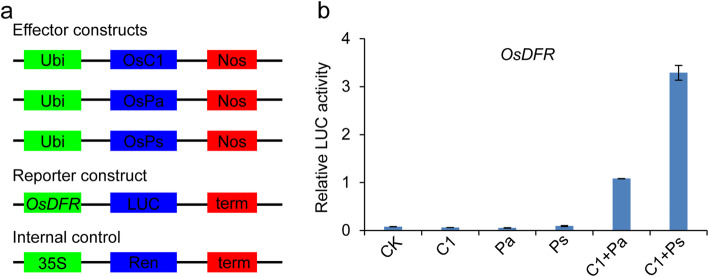
Both OsC1 and OsPa or OsPs were necessary for the transcriptional expression of *OsDFR* promoter. **a** Schematic representation of the effector and reporter constructs. Full-length coding regions of *OsC1*, *OsPa* and *OsPs* under control of the ubiquitin promoter were used as the effectors. Firefly luciferase gene *LUC* driven by the *OsDFR* promoters and the Renilla luciferase gene *Ren* driven by the 35S promoter were used as the reporter and internal control, respectively. **b** Transient dual-luciferase assays were performed in *Nicotiana benthamiana* leaves to investigate the effects of OsC1, OsPa and OsPs on the transcriptional expression of *OsDFR*. Relative LUC activity was measured by the ratio Firefly luciferase (LUC): Renilla luciferase (REN), and data are presented as means ± SD (*n* = 3)

We next performed dual-luciferase (LUC) assays in *N. benthamiana* leaves to test the effects of OsC1, OsPa and OsPs on transcriptional expression of *OsDFR*. As indicated in Fig. 6a, b, OsC1, OsPa or OsPs each alone could barely activate *OsDFR*, however, co-expression of OsC1 and OsPa or OsC1 and OsPs significantly activated *OsDFR*, indicating that activation of *OsDFR* depended on formation of OsC1-OsPa or OsC1-OsPs complexes (Fig. 6b). The OsC1-OsPa or OsC1-OsPs complex could also initiate expression of other anthocyanin biosynthesis genes, such as *CHS*, *CHI*, *F3’H*, *F3H* and *ANS* (Fig. S10). We postulated that OsPa or OsPs as a bHLH partner of OsC1 (R2R3-MYB) is required for activation of *OsDFR* and other structural genes for OsC1-dependent anthocyanin biosynthesis, finally determining specific anthocyanin accumulation in apiculi or stigmas.

### Functional *OsPa* and *OsPs* Potentially Pre-Exist in Almost all Natural Rice Accessions

Previous studies revealed that *OsC1* and *OsDFR* played determinant roles in evolution of the anthocyanin biosynthesis pathway (Sun et al. 2018; Zheng et al. 2019). Here, we identified tissue-specific genes, *OsPa* and *OsPs*, which also played crucial roles for purple-colored pigmentation in apiculi and stigmas, respectively. To comprehensively decipher color diversification patterns in rice, we analyzed sequence variations in the CDSs of *OsPa* and *OsPs* in a panel of 234 rice accessions including the sequencing reference variety Nipponbare, 175 varieties from the mini-core collection (Zhang et al. 2011) and 58 varieties carrying purple apiculi and stigmas (Table S4).

**Fig. 7:**
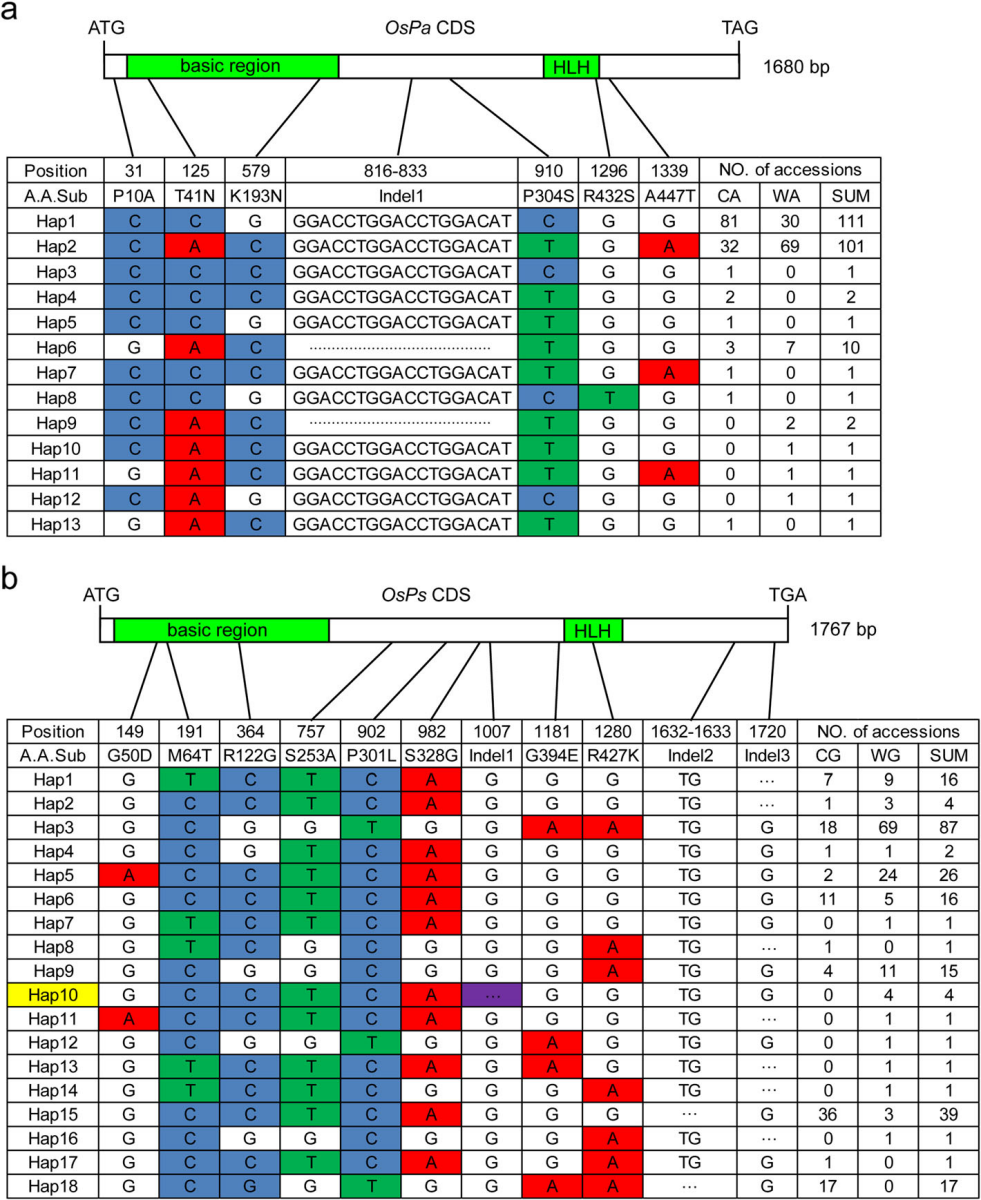
Haplotype analysis of *OsPa* and *OsPs* in a nature rice panel. Haplotype analysis of *OsPa* (**a**) and *OsPs* (**b**) in 234 rice accessions. A.A.Sub are amino acid substitutions. CA, colored apiculi; WA, straw-white apiculi; CG, colored stigmas; WG, straw-white stigmas. Deletion and insertion sites are indicated by dashed lines. Haplotype analysis of *OsPa* and *OsPs* was carried out with reference to the sequences of Nipponbare (Hap1). A non-functional haplotype is indicated in yellow. Polymorphic nucleotides causing loss-of-function are in purple. The number of accessions for each haplotype is shown in the right column

For *OsPa*, seven natural variations including six nonsynonymous single nucleotide polymorphisms (SNPs) and one 18 bp InDel were detected in the panel of 234 accessions (Fig. 7a; Table S4), and all were distributed in both the accessions with purple or brown apiculi and those with straw-white apiculi (Table S4), indicating that none of these variations affected the function of *OsPa*. Thirteen *OsPa* haplotypes were defined based on these seven variations (Fig. 7a); among them *Pa*-Hap1 and *Pa*-Hap 2 were prevailed at frequencies of 47.44 and 43.16%, respectively, followed by *Pa*-Hap 6 with a frequency of 4.27%. The other 10 haplotypes were quite rare with frequencies ranging from 0.43 to 0.85%. The three prevalent haplotypes (95.28%) were distributed in both the accessions with purple or brown apiculi and those with straw-white apiculi (Fig. 7a; Table S4). Therefore, we deduced that functional *OsPa* allele could pre-exist in almost all the natural rice accessions.

For *OsPs*, a total of eleven variations including eight nonsynonymous SNPs and three InDels were detected, and among them only the InDel1 variation caused premature termination of translation leading to loss of function of *OsPs*, and existed in only four accessions with straw-white stigmas (Aijiaonante, Jinzhinuo, Sankecun and Haoxiang). Due to their distribution in both pigmented and non-pigmented accessions it was clear that the other 10 variations did not affect stigma color (Fig. 7b; Table S4). Eighteen *OsPs* haplotypes were defined based on these 11 variations (Fig. 7b; Table S4). Among them, *Ps*-Hap3 was the most prevalent haplotype at 37.18%, *Ps*-Hap15, *Ps*-Hap5, *Ps*-Hap18, *Ps*-Hap1, *Ps*-Hap6 and *Ps*-Hap9 were moderately prevalent haplotypes with occurrence frequencies ranging from 6.41 to 16.67%, and the other 11 haplotypes were rare (0.43 to 1.71%). Except *Ps*-Hap10, which was the haplotype carrying the Indel1 variation, all other haplotypes except the five extremely rare ones, *Ps*-Hap11–14 and *Ps*-Hap16 (accounting for 2.1%), included accessions with pigmented stigmas (Fig. 7b; Table S4). We again deduced that the functional *OsPs* allele could pre-exist in the majority of rice accessions.

In order to investigate the roles of promoter sequence variations in the specific expression and regulation of the transcription factors, we sequenced the 2.0 kb promoter regions of *OsPa* and *OsPs* and analyzed their sequence variations in the panel of 234 rice accessions (Table S5). As a result, 37 variations including16 for the *OsPa* promoter region (13 types) and 21 for the *OsPs* promoter region (11 types) were detected, and all were distributed in both pigmented and non-pigmented accessions (Fig. S11a, b; Table S5). The result indicated that none of these variations caused functional differences of *OsPa* and *OsPs* between pigmented and non-pigmented rice accessions, and thus there might not be sequence-specific expression elements in the promoter regions of *OsPa* and *OsPs* that regulated their specific expression.

### Correlation of Haplotype Combinations of *OsC1*, *OsDFR*, *OsPa* and *OsPs* with Apiculus and Stigma Color Variations in Rice Accessions

**Fig. 8:**
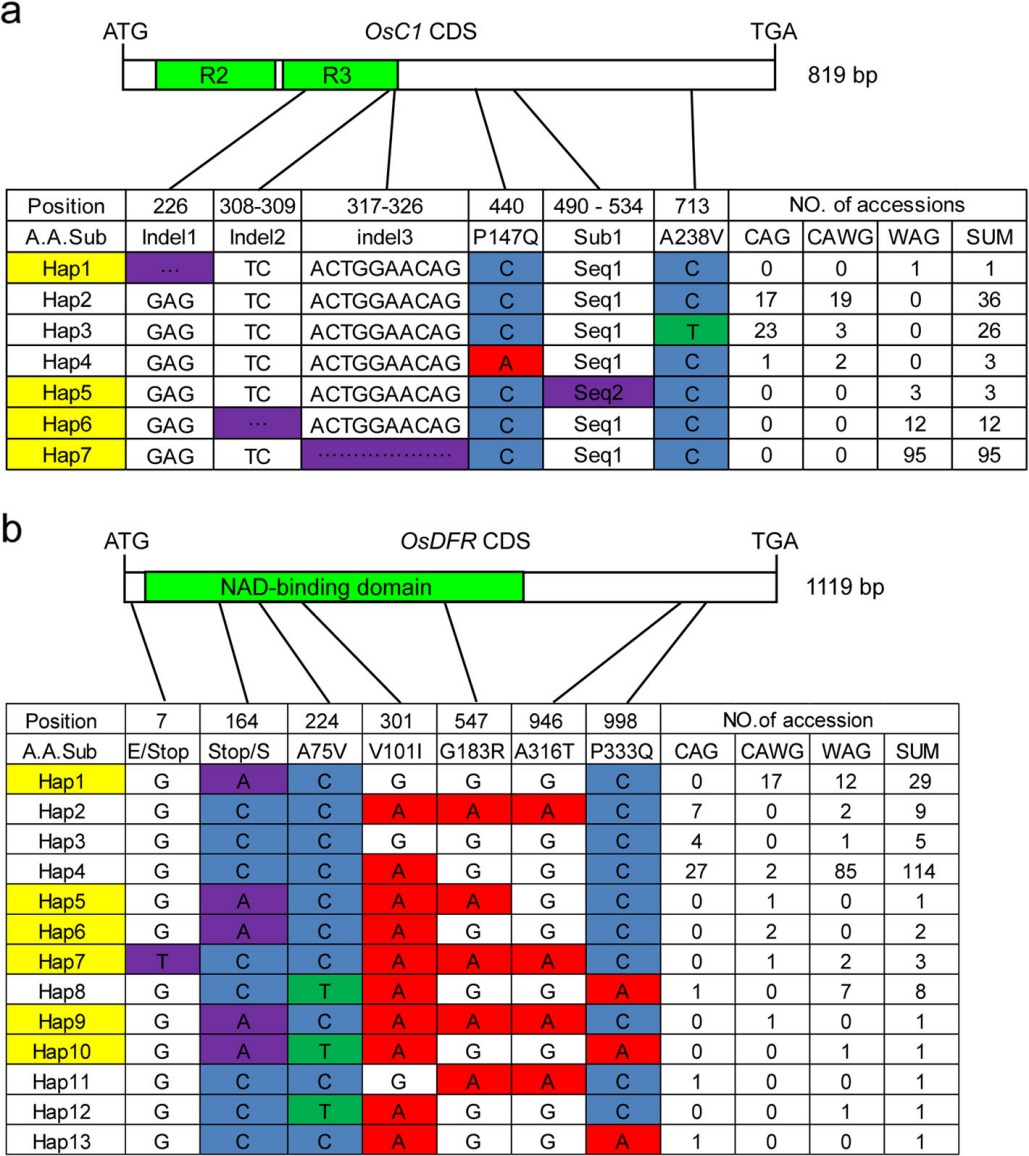
Haplotype analysis of *OsC1* and *OsDFR* in a panel of 176 rice accessions. Haplotype analysis of *OsC1* (**a**) and *OsDFR* (**b**) in the mini core collection. A.A.Sub, indicates amino acid substitution. CAG, colored apiculi and colored stigmas; CAWG, colored apiculi and straw-white stigma; WAG, straw-white apiculi and straw-white stigma. Deletion and insertion sites are indicated by dashed lines. Haplotype analyses of *OsC1* and *OsDFR* were conducted according to the sequences of Nipponbare (Hap1). Non-functional haplotype is in yellow. Polymorphic nucleotides causing loss-of-function are in purple. The number of accessions of each haplotype is shown in the right column. Sub1, substitution of ACGGCAGCGGCGGCGGCGGCGGCGACGACGA CCACCGTGTGGGCG (Seq1) by GCAGCCAGCCT (Seq2)

Phenotypic analyses of the 176 rice varieties showed that 23.29% (41 accessions) possessed purple apiculi and purple stigmas, 13.64% (24) possessed brown/red apiculi but straw-white stigmas, and 63.07% (111 accessions) had straw-white apiculi and straw-white stigmas (Tables S4, S5). In order to investigate the correlation between the color phenotypes and haplotype combinations of *OsC1*, *OsDFR*, *OsPa* and *OsPs*, we further analyzed sequence variations of *OsC1* and *OsDFR* in these accessions by sequencing their CDSs. For *OsC1*, two nonsynonymous SNPs, three InDels and a 45 bp substitution (Sub1) were detected, and three functional haplotypes (*C1*-Hap2–4) and four non-functional haplotypes (*C1*-Hap1 and *C1*-Hap5–7) were identified (Fig. 8a; Table S6). For *OsDFR*, seven nonsynonymous SNPs were detected, and seven functional haplotypes (*DFR*-Hap2–4, *DFR*-Hap8 and *DFR*-Hap11–13) and six non-functional haplotypes (*DFR*-Hap1, *DFR*-Hap5–7 and *DFR*-Hap9–10) were identified (Fig. 8b; Table S6).

The 176 accessions were genotypically classified into six groups by functional or non-functional alleles of *OsC1*, *OsDFR*, *OsPa* and *OsPs*. As expected, all the 41 accessions in Group I had purple apiculi and purple stigmas, the two in Group II had purple apiculi but straw-white stigmas, all 22 in Group III had brown/red apiculi but straw-white stigmas, whereas accessions in Groups IV, V and VI had no pigment in both apiculi and stigmas (Tables S7, S8). These data corroborated the results of the genetic dissection of purple apiculi and stigmas (Figs. S3, S4) and those of the functional validation of *OsC1*, *OsDFR*, *OsPa* and *OsPs* (Figs. 2, 3, Fig. S6). We thus concluded: 1) in the presence of *OsPa*, apiculi displayed purple color when both *OsC1* and *OsDFR* were present, and the color changed to red or brown color when *OsC1* was present but *OsDFR* was absent. There was no pigmentation regardless of the presence of *OsDFR* when OsC1 was absent. In the absence of *OsPa*, the apiculi displayed no color regardless of the presence of both *OsC1* and *OsDFR*; 2) stigmas were purple only when all three genes *OsC1*, *OsDFR* and *OsPs* were present, and the absence of any one resulted in no pigmentation.

## Discussion

### Determinant Factors for Anthocyanin Biosynthesis in Rice Apiculi and Stigmas

The *C*-*A*-*P* gene system controlling anthocyanin coloration was firstly established for purple apiculi of *japonica* rice (Takahashi 1957; Kondo 1963). In this system, two basic complementary genes, *C* and *A*, were assumed to be responsible for the production of anthocyanin color together with a *P* gene that conferred color to specific organs. The genetics of purple apiculi and purple stigmas are examples of traits that until now were largely unknown.

In the present study, we first mapped and isolated complementary genes *OsC1* and *OsDFR* responsible for the purple coloration of apiculi and stigmas in *indica* cv. XQZ (Fig. 1, Fig. S3; Table S1). Introduction of *OsC1* alone with its native promoter into cv. Kitaake caused brown apiculi but straw-white stigmas in a manner similar to that reported by Zhao et al. (2016), but in combination with *OsDFR* it produced not only purple apiculi but also purple stigmas (Fig. 2, Fig. S4). We then identified the tissue-specific pigmentation genes, *OsPa* and *OsPs* from shortlisted candidates by means of the phylogenetic analysis of all anthocyanin biosynthesis-associated bHLH TFs in maize and rice and CRISPR/Cas9 knockout (Fig. S5; Table S2). Knockout of *OsPa* in both XQZ and LTH backgrounds caused loss of purple apiculus but retention of purple stigmas, whereas knockout of *OsPs* produced the opposite effect (Fig. 3, Fig. S6). In addition, qRT-PCR revealed that that *OsPa* was strongly expressed in apiculi with an expression level at least 32-fold higher than that in stigmas, and on the contrary *OsPs* was strongly expressed in stigmas with an expression level at least 42-fold higher than that in apiculus (Fig. 4c, d). These results provided strong evidence that *OsPa* and *OsPs* acted as tissue-specific genes and participated in the pigmentation of apiculi and stigmas, respectively. Thus, purple pigmentation of each of apiculi and stigmas was indeed controlled by at least three genes, i.e. *OsC1*, *OsDFR* and *OsPa* or *OsPs*, which respectively corresponded to alleles *C*, *A* and *P* in the *C*-*A*-*P* gene system.

Based on sequencing and functional allele analysis of *OsC1*, *OsDFR*, *OsPa* and *OsPs*, we genotyped a panel of 176 rice accessions including the sequenced reference Nipponbare and 175 accessions from the Chinese mini-core collection for their apiculus and stigma pigmentation, and grouped them into six genotype groups (I-VI) based on apiculus and stigma color (Tables S7, S8). This confirmed the general applicability of the *OsC1*-*OsDFR*-*OsPa* and *OsC1*-*OsDFR*-*OsPs* systems to natural rice accessions regardless of subspecies for deciphering the regulatory mechanism of anthocyanin biosynthesis in apiculi and stigmas.

### The Regulatory Systems of Anthocyanin Biosynthesis in Rice Apiculi and Stigmas

**Fig. 9:**
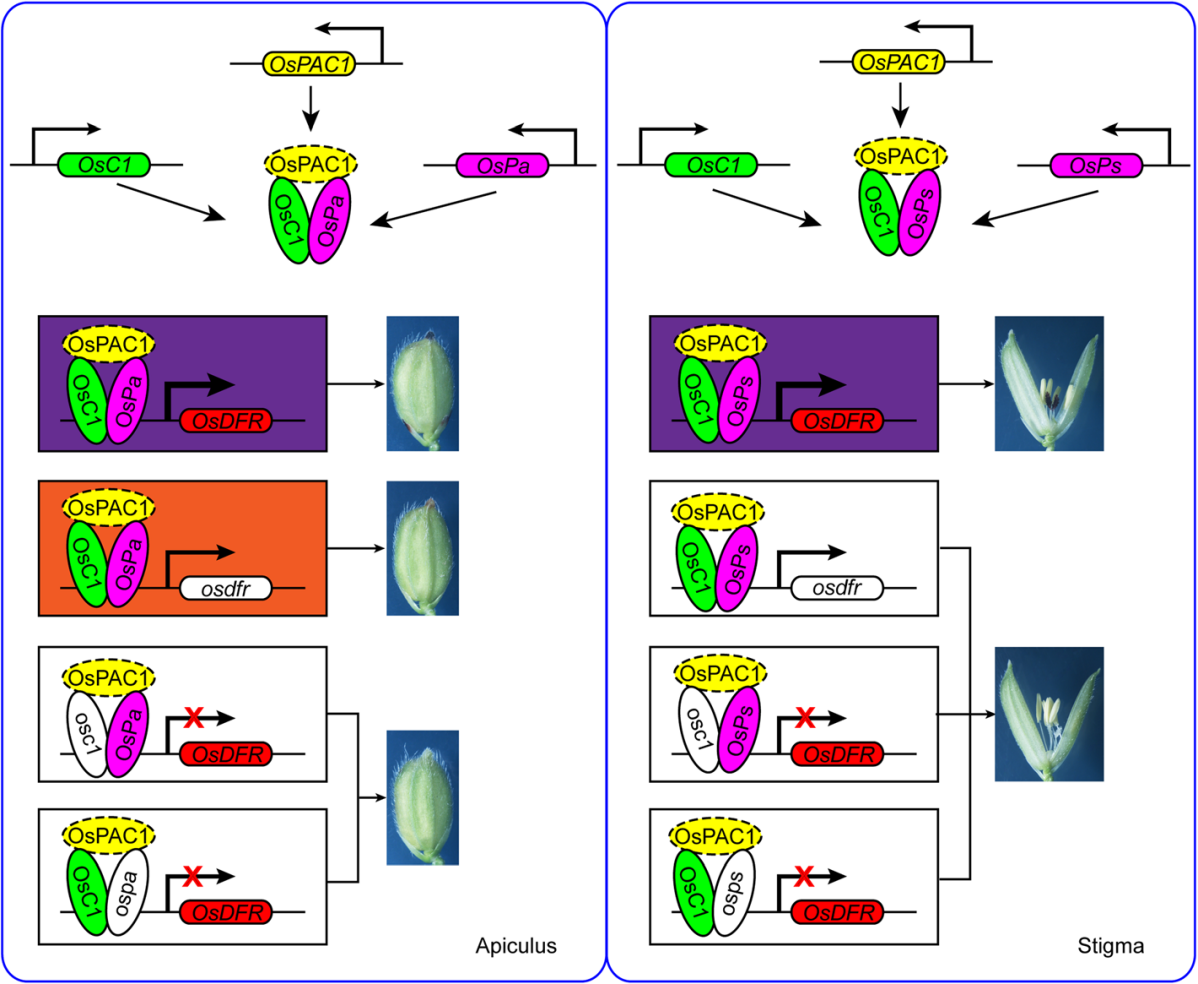
Working models of apiculus and stigma coloration in rice. Both *OsC1* and *OsDFR* had extremely high expression levels in both apiculus and stigmas, whereas *OsPa* and *OsPs* were more strongly expressed in the respective apiculi and stigmas. When all of *OsC1*, *OsDFR*, *OsPa* and *OsPs* were functional the OsC1-OsPa-OsPAC1 and OsC1-OsPs-OsPAC1 complexes activated expression of *OsDFR* resulting in purple apiculi and purple stigmas, respectively. When *OsC1* was not functional, it affected complex formation, therefore decreasing the expression of *OsDFR* and leading to non-pigmented apiculi and stigmas. When *OsDFR* was not functional, there was no stigma color and apiculi were brown. When *OsPa* or *OsPs* were nonfunctional, both apiculi and stigmas were non-pigmented. OsC1, OsPa and OsPs represented functional proteins corresponding to the products of *OsC1*, *OsPa* and *OsPs*, respectively. osc1, ospa and osps represented nonfunctional products of the *OsC1*, *OsPa* and *OsPs* loci, respectively. *OsDFR and osdfr* represented functional and nonfunctional *OsDFR*, respectively. Solid ellipse, interaction of the protein inside with the other had been confirmed; dashed ellipse, interaction of the protein inside with the other needed to confirm

The *OsC1*-*OsDFR*-*OsPa* and the *OsC1*-*OsDFR*-*OsPs* gene systems are summarized in Fig. 9. The two systems share similar regulatory mechanisms of pigmentation as the *C*-*A*-*S* and the *OsC1*-*OsRb*-*OsDFR* gene models (Sun et al. 2018; Zheng et al. 2019). That is, the R2R3-MYB TF OsC1 (i.e. C1) interacts with tissue-specific bHLH TFs (OsPa, OsPs, S1/OsB2, or OsRb) to activate and elevate the expression levels of *OsDFR* (i.e. *A1*) and other anthocyanin biosynthesis genes, causing purple color in apiculi, stigmas, hulls or leaves, respectively.

Previous studies identified several putative tissue-specific genes for anthocyanin biosynthesis including *OsB1* (*Ra1*), *OsB2*, *Rb1*, and *Rb2* (Hu et al. 1996, 2000; Sakamoto et al. 2001). Only two, *OsB2* (i.e. *Kala4* and *S1*, hull-specific) and (i.e. *OsRb*, leaf-specific), had been functionally confirmed, and they encoded typical bHLH-type TFs sharing higher homology to OsPa (LOC_Os04g47040) than to OsPs (LOC_Os04g47080) (Fig. S5; Sun et al. 2018; Zheng et al. 2019). Nevertheless, *OsPa*, *S1* and *OsPs* are located in the same gene cluster on chromosome 4 sharing highly conserved basic regions and HLH domains. *OsPs*, formerly named *Ra1* and *OsB1*, had high homology along its entire length with the maize *R* (*Lc*) gene (Hu et al. 1996, 2000; Sakamoto et al. 2001). *OsPs* and its adjacent *S1* were earlier reported to participate in pigmentation of rice leaves (Sakamoto et al. 2001). However, the recent study of Zheng et al. (2019) suggested that both *OsPs* and *S1* might not be connected with anthocyanin biosynthesis due to their extremely low expression levels in leaves. On the contrary, *OsPs* is most strongly expressed in stigmas and functions as a stigma-specific gene for anthocyanin biosynthesis (Figs. 3a, 4d, Fig. S6a), whereas *S1* was still regarded as a determinant of purple hull although a ‘gain-of-function’ mutation in its promoter region is responsible for purple pericarp (Oikawa et al. 2015; Sun et al. 2018). To our knowledge this is the first report on *OsPa* and its function in anthocyanin biosynthesis as an apiculus-specific gene. The fact that the clustered bHLH-type TF homologs *OsPa*, *S1* and *OsPs*, have different tissue-specific functions could reflect the allelic constitutions at the *OsPa*/*S1*/*OsPs* regulatory loci, and may provide cues for understanding the functional diversification mechanisms of tissue-specific regulators related to anthocyanin biosynthesis in plants.

The two types of TFs in maize, a R2R3-MYB-related protein and a bHLH-containing protein, interact with each other and activate anthocyanin biosynthesis genes as a single complex (Petroni and Tonelli 2011). Our results showed that R2R3-MYB-type regulator OsC1 interacts with two bHLH-type TFs, OsPa and OsPs, respectively in determining the expression of *OsDFR* and some other structural genes (Figs. 5, 6, Fig. S10), leading to the tissue-specific distribution of purple pigmentation. In Arabidopsis, the WD40 repeat protein TTG1 interacts with R2R3-MYB-type TFs and bHLH-type TFs to form MYB-bHLH-WD40 complexes with roles in anthocyanin accumulation in vegetative tissues or proanthocyanin accumulation in developing seeds. The mutation of TTG1 directly leads to the appearance of yellow seed coat, and the proanthocyanin of seed coat cannot be synthesized (Xu et al. 2015). In tobacco, the transformation of anthocyanin-related R2R3-type MYB transcription factor activates the synthesis of anthocyanins that are inseparable from the expression of *WD40* (Montefiori et al. 2015). *PAC1*, the homolog of *TTG1* in maize, is required for the anthocyanin accumulation in the pericarp (Petroni and Tonelli 2011). In rice, Sun et al. (2018) assumed that *OsPAC1* was not essential for anthocyanin biosynthesis because there were no functional mutations in natural rice germplasm. However, Zheng et al. (2019) assumed that *OsPAC1* was required for full activation of anthocyanin biosynthesis genes by interacting with OsC1 and OsRb, and Zhu et al. (2017) found that the specific biosynthesis of anthocyanins in rice endosperm was involved in up-regulation of endogenous genes *OsWD40*. In the present study, we found that *OsPAC1* was expressed constitutively in all tissues tested (Fig. S12), and no any functional mutations in the *OsPAC1* CDS occurred between the pigmented and non-pigmented rice accessions (Table S9). Therefore, we deduced that functional *OsPAC1* allele could pre-exist in almost all the natural rice accessions, and be indispensable for anthocyanin biosynthesis in rice apiculi and stigmas.

In order to decipher rice color diversification patterns, we investigated the CDS variations of *OsPa* and *OsPs* in a panel of 234 natural rice accessions (Table S4). All seven variations in *OsPa* and 10 of 11 variations (except InDel1) of *OsPs* did not affect their normal functions we deduced that all *OsPa* haplotypes and most *OsPs* haplotypes in the panel functioned normally (Tables S7, S8), perhaps explaining why these two genes were not characterized by conventional genetics and previous map-based cloning studies (Han et al. 2006; Fan et al. 2008; Chen et al. 2010; Zhao et al. 2016). It also addresses the fact that most rice lines developed from crosses express anthocyanin color in the stigma only when the apiculus is colored (Takahashi 1964). Oka (1991) reported an upland variety Gaisen-mochi with non-pigmented apiculi and purple stigmas, and postulated a recessive inhibitor gene *i-Ps1* in addition to the genotype *C A p Ps-1*. According to the *OsC1-DFR-OsPa* and *OsC1-OsDFR-OsPs* systems we predict that Gaisen-mochi has genotype *OsC1-DFR-Ospa*-*OsPs*, in which loss of function of *OsPa* is responsible for the straw-white apiculus but does not affect the coloration of stigmas. Reddy (1996) reported a leaf blade-specific dominant inhibitor of anthocyanin pigmentation (*Ilb*) in *indica* lines N22B and N22W, that inhibited pigmentation of the leaf blade. It was observed that some crosses between pigmented and non-pigmented rice lines produce non-pigmented F_1_ plants, suggesting a common presence of dominant inhibitor alleles among rice cultivars (Reddy 1996). Recently, a number of repressor proteins including R3-MYB and R2R3-MYB repressors that limit expression of anthocyanin biosynthesis genes were identified in horticultural plants (Albert et al. 2014; Naing and Kim 2018; Zhang et al. 2020), strengthening the need to search for tissue-specific inhibitors of anthocyanin biosynthesis in rice.

### Brown Apiculi Color May Be Caused by the OsC1-OsPa Complex Rather than OsC1 Alone

*OsC1* alone was regarded as the chromogen gene responsible for the brown or red apiculi (Saitoh et al. 2004; Fan et al. 2008; Zhao et al. 2016; Sun et al. 2018). However, our CRISPR-Cas9 knockout results for *OsPa* demonstrated that *OsC1* itself does not produce color when functioning alone (Fig. 3b, Fig. S6b), but produces brown apiculi only when functioning together with *OsPa* (Figs. 6, 7, Fig. S10). This indicates that *OsC1* might not be a chromogen gene for anthocyanin, but could work as a switch in controlling color production as previously reported (Zhao et al. 2016; Sun et al. 2018).

The reasons why the *OsC1*-complementation and/or transgenic overexpression lines in Kitaake and Nipponbare backgrounds produced brown or red color only in the apiculi in previous studies (Zhao et al. 2016; Sun et al. 2018) could be that both possessed the genotype *Osc1*-*Osdfr*-*OsPa*-*OsPs* (Figs. 1, 2; Table S7). When *OsC1* alone was introduced into HC1 with genotype of *Osc1*-*S1*-*osdf*, the hull color of all transgenic lines was brown (Sun et al. 2018), indicating that the OsC1-S1 complex rather than OsC1 alone caused brown hulls. The brown color of rice hulls involves accumulation of the main products as flavonols and flavanones in the absence of a functional *OsDFR* (Sun et al. 2018). Thus, we speculate that brown apiculi could have a similar control mechanism as brown hulls. Further work including identification of the catalytic enzyme targeted by the OsC1-OsPa complex and verification of the interaction between this enzyme and the OsC1-OsPa complex is needed to determine the regulatory systems of flavonol and/or flavanone biosynthesis in brown apiculi.

## Conclusion

In the present study, we identified four determinant factors for purple apiculi and stigmas from *indica* cultivar Xieqingzao and dissected their regulatory mechanisms by genetic and molecular approaches. Among these determinants, *OsC1* encodes a R2R3-MYB transcriptional factor, *OsDFR* encodes a dihydroflavonol 4-reductase, and *OsPa* and *OsPs* encode bHLH transcription factors that function as apiculus- and stigma-specific regulator, respectively. OsC1 specifically interacts with OsPa or OsPs to activate *OsDFR* and other anthocyanin biosynthesis genes, resulting in purple-colored apiculi or stigmas. *OsC1* does not produce color when functioning alone, but produces brown apiculi only when functioning together with *OsPa*, indicating that *OsC1* itself might not be a chromogen gene for anthocyanin. Genotyping and phenotyping of 176 rice accessions validated the general applicability of the *OsC1*-*OsDFR*-*OsPa* and *OsC1*-*OsDFR*-*OsPs* models to natural rice accessions regardless of subspecies. Our findings disclosed the biological functions of *OsC1*, *OsPa* and *OsPs*, and shed light on the specific regulatory systems of anthocyanin biosynthesis in apiculi and stigmas, a further step in understanding the regulatory network of anthocyanin biosynthesis in rice.

## Materials and Methods

### Plant Materials and Growth Conditions

*Indica* cv. XQZ (female parent) with purple apiculi and purple stigmas and *japonica* cv. Kitaake (male parent) with straw-white apiculi and stigmas were crossed to develop F_1_ and F_2_ populations for genetic analysis and gene mapping. LTH with purple apiculus and stigma was used for knockout analysis of tissues-specific genes. A panel of 234 rice accessions, including Nipponbare, 175 varieties from the mini-core collection and 58 varieties carrying purple apiculi and stigmas were used for haplotype analysis of *OsC1*, *OsDFR*, *OsPa* and *OsPs* (Table S4). All seeds were seeded in an incubator and 30-day-old seedings were transplanted and grown in a paddy field at the Institute of Crop Science Shunyi Experimental Station in Beijing during April–October.

### Extraction and Quantification of Anthocyanin

Fifty milligrams of apiculi and stigmas respectively were isolated from rice florets 1 day post heading. Total anthocyanins from apiculi and stigmas were extracted and quantified as previously reported (Mehrtens et al. 2005). All samples were measured with three biological replicates.

### Positional Cloning of *OsC1* and Os*DFR*

To map the target genes determining apiculus and stigma color, 212 InDel markers covering all the 12 chromosomes (Wang et al. 2017) were used for initial screening for polymorphisms between XQZ and Kitaake. Molecular markers used for fine mapping were designed based on the nucleotide polymorphisms between *japonica* cv. Nipponbare and *indica* cv. 9311. Annotation of predicted ORFs (open reading frames) in the candidate region was based on the rice genome annotation project database (http://rice.plantbiology.msu.edu/cgi-bin/gbrowse/rice/).

### Vector Construction and Rice Transformation

A 4.8 kb genomic fragment of *OsC1* consisting of a 2.5 kb promoter region, the entire *OsC1* coding region, and a 1.0 kb downstream region was amplified from XQZ with the primer pairs 1305-C-F/1305-C-R and inserted the *Sal*I and *Afl*II restriction sites of the pCAMBIA1305.1 vector to generate transformation plasmid *proOsC1:OsC1* which was then introduced into calli of Kitaake via *Agrobacterium*-mediated transformation. A 5.9 kb DNA fragment of *OsDFR* covering the entire coding region plus 2390 bp upstream and 1921 bp downstream regions was amplified using primers 2300-DFR-F/R and inserted *EcoR*I/*Smal*I restriction sites of the binary vector pCAMBIA2300 to generate a fused *proDFR:DFR* construct which was introduced into calli of Kitaake and transgenic plants that already carried the the above 4.8 kb genomic fragment of *OsC1.*

To knock out six tissue-specific genes (Table S2) 18 or 20 bp gene-specific spacer sequences were cloned into the vector sgRNA-Cas9 (Duan et al. 2019) and subsequently introduced into the calli of XQZ and LTH via *Agrobacterium*-mediated transformation.

### Structure Analysis and Multiple Sequence Alignment

The functional domains of OsC1, OsPa and OsPs were analyzed using the Simple Modular Architecture Research Tools (SMART) program (http://smart.embl-heidelberg.de/smart/set_mode.cgi?NORMAL=1). Multiple sequence alignment was conducted by DNAMAN. The Gramene database (http://www.gramene.org/) were accessed to search for rice homologs of maize *R* (*B-peru*).

### Subcellular Localizations of OsC1, OsDFR, OsPa and OsPs

For subcellular localization, coding sequences (CDSs) of *OsC1*, *OsDFR*, *OsPa* and *OsPs* without stop codons were amplified and recombined into N-terminal of GFP in the pAN580 vector under the control of the CaMV 35S promoter. Fusion expression vectors were co-transformed into rice protoplasts with the nuclear marker D53-mCherry or empty-mCherry. Laser confocal scanning microscope (ZEISS Microsystems LSM 700) was used to detect fluorescence signals.

### RNA Extraction and qRT-PCR Analysis

RNA was extracted from young roots of 10-day-old seedlings, stems, flag leaves, hulls, leaf sheaths, apiculi and stigmas at heading using a ZR Plant RNA MiniPrep Kit (Zymo Research) following the manufacturer’s instructions. Reverse transcription and qRT-PCR were conducted as described previously (Wang et al. 2017) The rice *Ubiquitin* gene (*LOC_Os03g13170*) was used as an endogenous control, and the 2^-ΔΔCT^ method was used to evaluate relative levels of gene expression (Ma et al. 2019). The primer used for qRT-PCR was designed with GenScript (https://www.genscript.com/ssl-bin/app/primer).

### Yeast Two-Hybrid Assays

The full length of *OsC1* coding region was cloned and inserted into *Eco*RI/*Xho*I restriction sites of prey vector pGADT7 and the entire coding regions of *OsPa* and *OsPs* were amplified and recombined into a vector pGBKT7 bait. Various combinations of prey and bait vectors were co-transformed into yeast strain AH109 (Clontech). After 3 days of growth on SD-Trp/−Leu plates at 30 °C, the interactions between baits and preys were determined on selective media (SD-Leu/−Trp/−His/−Ade) at 30 °C. All assays were performed with three repeats.

### LCI Assays

Protocols used for LCI assays were as published (Chen et al. 2008). The CDS of *OsC1* without stop codon was fused into *Bam*HI*/Sal*I restriction sites of pCAMBIA1300-nLUC to generate the recombined construct nLUC-C1 under the control of the CaMV35S promoter. The CDSs of *OsPa* and *OsPs* were amplified and cloned into the *Kpn*I*/Sal*I restriction sites of pCAMBIA1300-cLUC to generate the fused constructs cLUC-Pa and cLUC-Ps with the CaMV35S promoter. The recombinants were then introduced into *Agrobacterium tumefaciens* strain EHA105. The combined nLUC and cLUC constructs or corresponding empty vectors were co-infiltrated into in *N. benthamiana* leaves. The transient dual-luciferase image was captured using a low-light-cooled CCD imaging apparatus (Night SHADE LB 985 [Berthold] with Indigo software) 48–72 h after infiltration.

### BiFC Assays

For the BiFC assays, full-length of OsPa and OsPs proteins were fused with N-terminal YFP (nYFP) to generate Yn-Pa and Yn-Ps constructs, respectively. OsC1 was fused with C-terminal YFP (cYFP) to generate Yc-OsC1 construct. The combined constructs Yc-OsC1/Yn-Pa, Yc-OsC1/Yn-Ps, Yc-OsC1/Yn were transiently co-expressed in *N. benthamiana* leaves via *Agrobacterium*-mediated transformation. Fluorescence was observed by confocal microscopy (ZEISS Microsystems LSM 700) 2 days after transformation.

### Transactivation Activity Assays

An approximately 2.5 kb promoter region of *OsDFR* was amplified from XQZ genomic DNA and cloned into the pGreenII 0800-LUC vector to generate a *DFR*_*pro*_*-LUC* reporter construct. The full-length CDSs of *OsC1*, *OsPa* and *OsPs* were amplified and recombined into the *Bam*HI/*Kpn*I restriction sites in the pCUbi1390 vector to generate effector constructs. The combined reporter and effector plasmids were introduced into *Agrobacterium* strains *EHA105*, and then transiently co-expressed in *N. benthamiana* leaves as described previously (Waadt and Kudla 2008). The *luciferase* gene from *Renilla reniformis* (Ren) under control of the CaMV35S promoter was used as the internal control. LUC activity was calculated with a Promega Kit (E2920) following the manufacturer’s instructions 48–72 h after transformation and the relative LUC activity was represented by the ratio of LUC/Ren.

### Primers

All the primers used in the study are listed in Supplementary Table S10.

## Supplementary Information

**Additional file 1: Fig. S1.** Color phenotypes of *indica* cultivar XQZ. (**a**) Purple stigma development of XQZ 2, 1 and 0 days before heading (DBH). Bars, 2 mm. (**b**) Purple apiculi development of XQZ 2 and 0 days before heading (DBH), and 7 days after heading (DAH). Bars, 1 mm.

**Additional file 2: Fig. S2*.*** Relative anthocyanin contents of apiculi and stigmas one day post heading in XQZ and Kitaake. Data are means ± SD of three biological replicates (Student’s *t*-test: **P < 0.01).

**Additional file 3: Fig. S3*.*** Inheritance of apiculi and stigma coloration. (**a**) Kitaake with straw-white apiculi and stigma was crossed with XQZ with purple apiculi and stigma. (**b**) F_1_ individuals exhibited purple apiculi and stigmas similar to XQZ. (C) The F_2_ population segregated into three phenotypes fitting a 9:3:4 ratio (apiculi and stigmas both purple; apiculi brown, stigmas straw-white; apiculi and stigmas both straw-white).

**Additional file 4: Fig. S4*.*** Apiculus color development in Kitaake, XQZ and transgenic complemented lines. Apiculus color development of (**a**) Kitaake, (**b**) *OsC1*-transgenic complemented lines, (**c**) *OsC1* and *OsDFR-*transgenic complemented lines, and (**d**) XQZ. St1 to St6 are − 1, 1, 7, 14, 21, and 26 days post heading.

**Additional file 5: Fig. S5*.*** Phylogenetic tree of all rice bHLH transcriptional factors (TFs) and known maize bHLH TFs associated with anthocyanin biosynthesis. The genes in blue denote known maize bHLH TFs associated with anthocyanin biosynthesis. The genes highlighted in red represented are the rice bHLH TFs closest to the known maize bHLH TFs. The tree was constructed using MEGA 5.2 and bootstrapped with 1000 replicates.

**Additional file 6: Fig. S6*.*** Phenotypes of *HLH1* and *HLH2* knockout mutants in the LTH background. (**a**) the stigma color changed from purple to straw-white in *hlh1* mutants. Bar, 1 mm. (**b**) Purple apiculus color is lost in *hlh2* mutants. Bar, 1 mm. (**c**) Sequencing of the CRISPR/Cas9-targeted sites of *HLH1-* and *HLH2-* knockout lines. Plus (+) and minus (−) indicate base insertions (in blue) and deletions (by hyphen), respectively, relative to LTH. Gray boxes denote coding sequences of *HLH1* and *HLH2*, and blue boxes are the untranslated regions. *hlh1–2* and *hlh1–3* are independent *HLH1*-transgenic knockout lines. *hlh1–2* has a six-base deletion at position 52, causing a two-amino acids deletion. *hlh1–3* has a four-base deletion at position 54, causing a premature termination of translation. *hlh2–3* and *hlh2–4* are independent *HLH2*-transgenic knockout lines. *hlh2–3* has a two-base deletion*,* and *hlh2–4* has a single base insertion, both causing premature termination of translation.

**Additional file 7: Fig. S7*.*** Protein structures of OsC1*,* OsPa, OsPs and physical distance analysis of among three bHLH transcriptional factors (TFs)*.* (**a-c**) Protein structures of OsC1, OsPa and OsPs. Numbers above the diagrams indicate residue positions. R2R3 repeats, basic regions and hydrophobic HLH regions are labelled with green boxes. (**d**) Physical distances separating three bHLH TFs on chromosome 4 indicated above the genes.

**Additional file 8: Fig. S8*.*** Amino acid sequence alignments of OsPa, OsPs and S1 determined using DNAMAN. Consensus amino acids are shown at the bottom with lowercase letters; black indicates 100% identify; green indicates > 50% identify; and yellow indicates > 33% identify. Red and blue lines indicate the basic region and HLH domain, respectively.

**Additional file 9: Fig. S9*.*** Subcellular localization of OsC1, OsPa, OsPs and OsDFR in rice protoplasts. The OsC1-, OsPa-, OsPs-GFP fusion proteins were transiently co-expressed with nuclear marker D53-mCherry in rice protoplasts. OsDFR-GFP fusion protein was co-expressed with empty-mCherry in rice protoplasts. Left to right: images of GFP (green), mCherry (red), protoplast, and merged GFP and mCherry.

**Additional file 10: Fig. S10***.* Transient activation assays of OsC1, OsPa and OsPs on the promoters of five structural genes. (**a**) Schematic representation of the effector and reporter constructs. Full-length coding regions of *OsC1*, *OsPa* and *OsPs* under control of the ubiquitin promoter were used as the effectors. The Firefly luciferase gene *LUC* driven by the five structural genes promoters and the Renilla luciferase gene *Ren* driven by the 35S promoter were used as reporter and internal control, respectively. (**b-f**) Transient dual-luciferase assays were performed in *Nicotiana benthamiana* leaves to investigate the effects of OsC1, OsPa and OsPs on the transcriptional expression of structural genes. Relative LUC activity was measured by Firefly luciferase (LUC): Renilla luciferase (REN) ratio and data are presented as mean ± SD (*n* = 3).

**Additional file 11: Fig. S11.** Sequencing of 2.0 kb promoter regions of *OsPa* and *OsPs* in a panel of 234 rice accessions. Promoter sequence variations of *OsPa* (a) and *OsPs* (b) in the 234 rice accessions. B. sub are base substitutions. CA, colored apiculi; WA, straw-white apiculi; CG, colored stigmas; WG, straw-white stigmas. Deletion and insertion sites are indicated by dashed lines. Promoter sequence analysis of *OsPa* and *OsPs* was carried out with reference to the sequences of Nipponbare (T1). Seq1, CTAAAAT; Seq2, ACGACACT. The numbers at the top represented the base variation positions from the start codon.

**Additional file 12: Fig. S12.** Expression analysis of *OsPAC1* in XQZ, LTH, Kitaake and Nipponbare (Nip). The samples were collected from roots of 10-day-old seedlings, stems, flag leaves, hulls, leaf sheaths, apiculi, and stigmas at heading. Data are presented as means ± SD (*n* = 3).

**Additional file 13: Table S1*.*** Segregation of apiculi and stigma color in the F_2_ population derived from a cross between Kitaake and XQZ.

**Additional file 14: Table S2*.*** Identification of tissue-specific bHLH-type genes in rice genome.

**Additional file 15: Table S3.** Amino acids sequences alignment of three bHLH transcription factors.

**Additional file 16: Table S4*.*** Haplotype analysis of *OsPa* and *OsPs* in 234 natural rice accessions.

**Additional file 17: Table S5.** Promoter sequence variations of *OsPa* and *OsPs* in a panel of 234 natural rice accessions.

**Additional file 18: Table S6.** Haplotype analysis of *OsC1* and *OsDFR* in 176 natural rice accessions.

**Additional file 19: Table S7.** Summary of the *OsC1*, *OsDFR*, *OsPa* and *OsPs* haplotypes in 176 natural rice accessions.

**Additional file 20: Table S8.** Analysis of haplotype combinations of the *OsC1*, *OsDFR*, *OsPa* and *OsPs* genes in lines with apiculus and stigma colors.

**Additional file 21: Table S9.** Analysis of *OsPAC1* coding region in a panel of 176 natural rice accessions.

**Additional file 22: Table S10*.*** Sequences of primers used in this study.

## Data Availability

The datasets supporting the conclusions of this article are included within the article and its additional files.
