# Supplementary figures and images for: Determinant Factors and Regulatory Systems for Anthocyanin Biosynthesis in Rice Apiculi and Stigmas
Source: Rice (N Y). 2021 Apr 21;14:37. doi: 10.1186/s12284-021-00480-1 (PMC8060382; doi:10.1186/s12284-021-00480-1)

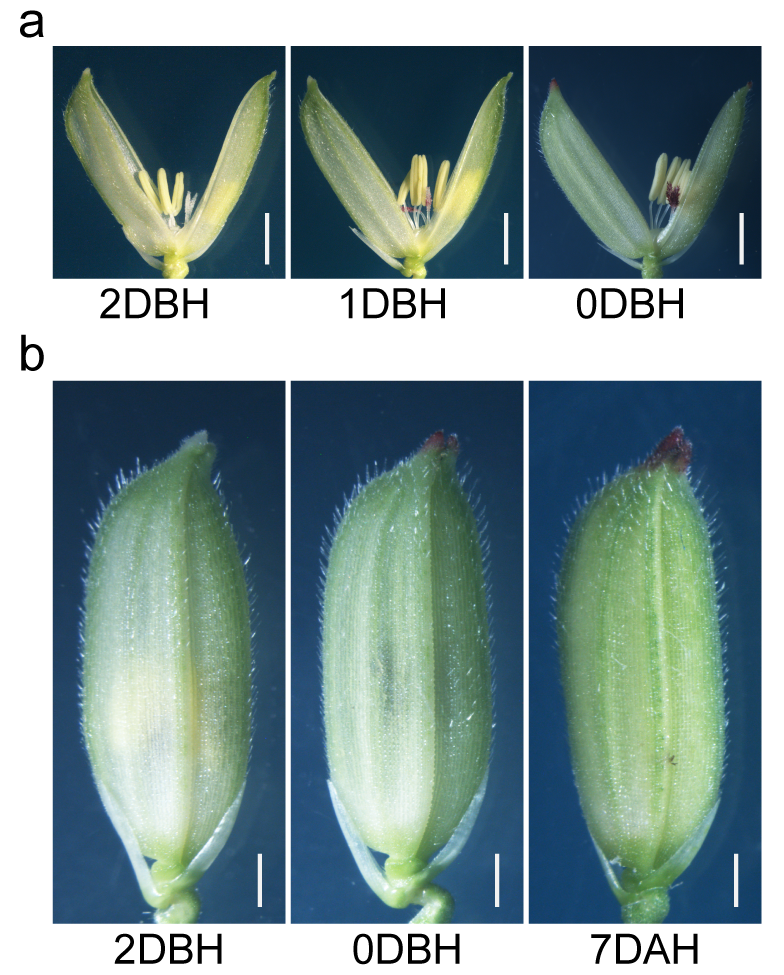

Supplement: Supplementary file 1 — Additional file 1: Fig. S1. Color phenotypes of indica cultivar XQZ. (a) Purple stigma development of XQZ 2, 1 and 0 days before heading (DBH). Bars, 2 mm. (b) Purple apiculi development of XQZ 2 and 0 days before heading (DBH), and 7 days after heading (DAH). Bars, 1 mm. [file 12284_2021_480_MOESM1_ESM.tif]

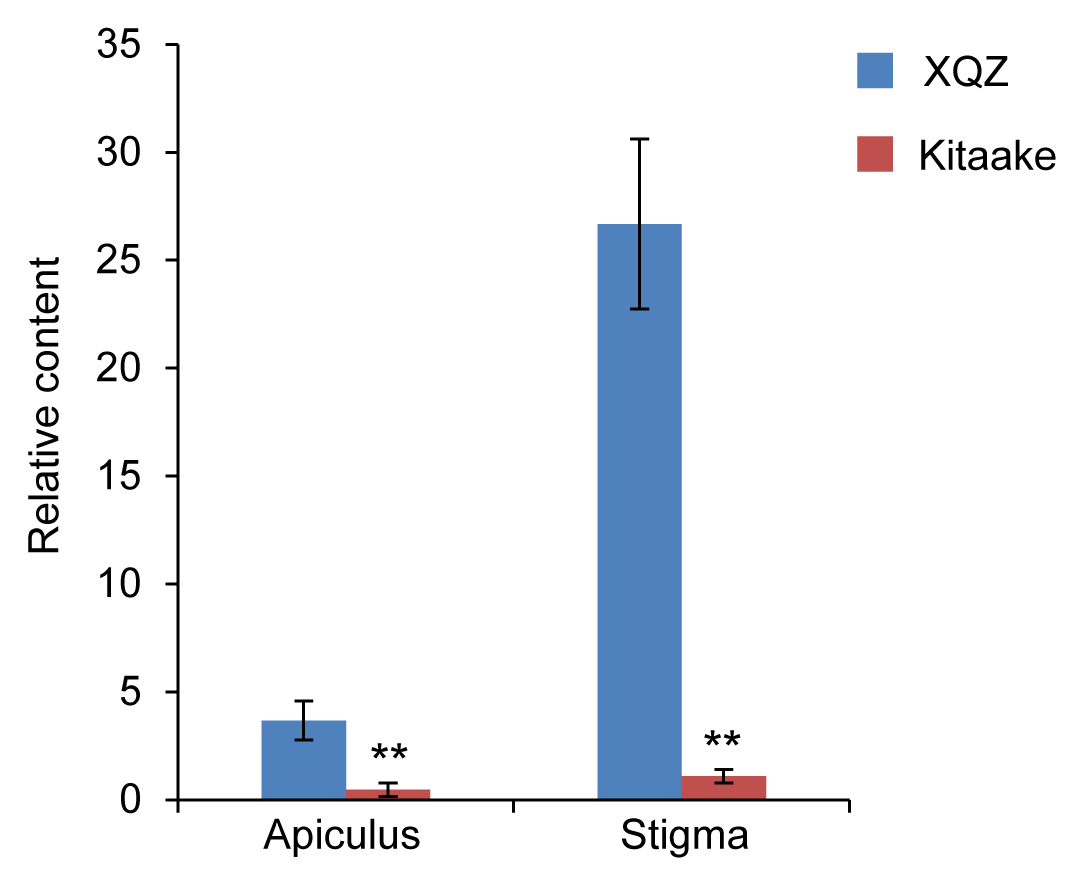

Supplement: Supplementary file 2 — Additional file 2: Fig. S2. Relative anthocyanin contents of apiculi and stigmas one day post heading in XQZ and Kitaake. Data are means ± SD of three biological replicates (Student’s t-test: **P < 0.01). [file 12284_2021_480_MOESM2_ESM.tif]

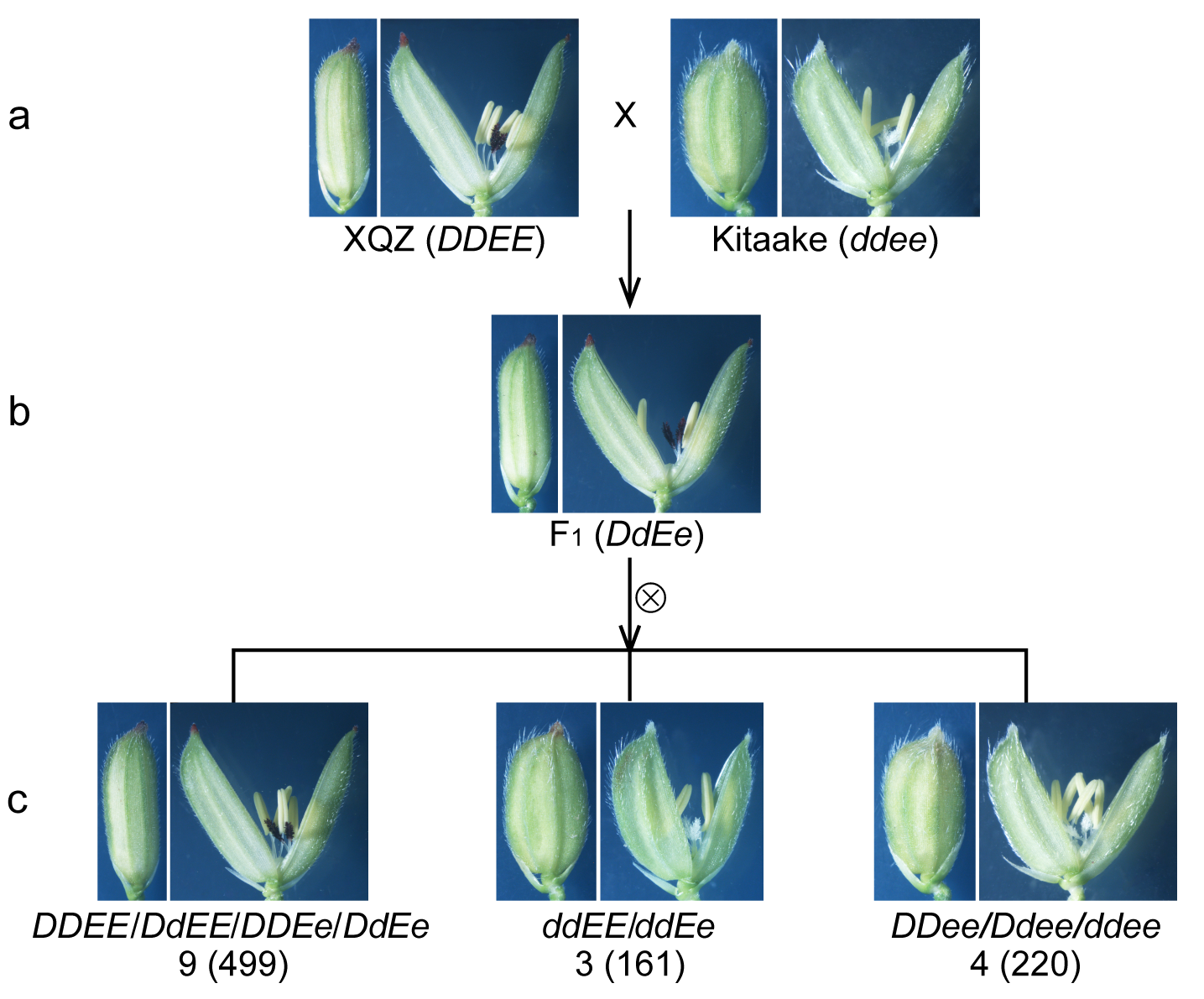

Supplement: Supplementary file 3 — Additional file 3: Fig. S3. Inheritance of apiculi and stigma coloration. (a) Kitaake with straw-white apiculi and stigma was crossed with XQZ with purple apiculi and stigma. (b) F1 individuals exhibited purple apiculi and stigmas similar to XQZ. (C) The F2 population segregated into three phenotypes fitting a 9:3:4 ratio (apiculi and stigmas both purple; apiculi brown, stigmas straw-white; apiculi and stigmas both straw-white). [file 12284_2021_480_MOESM3_ESM.tif]

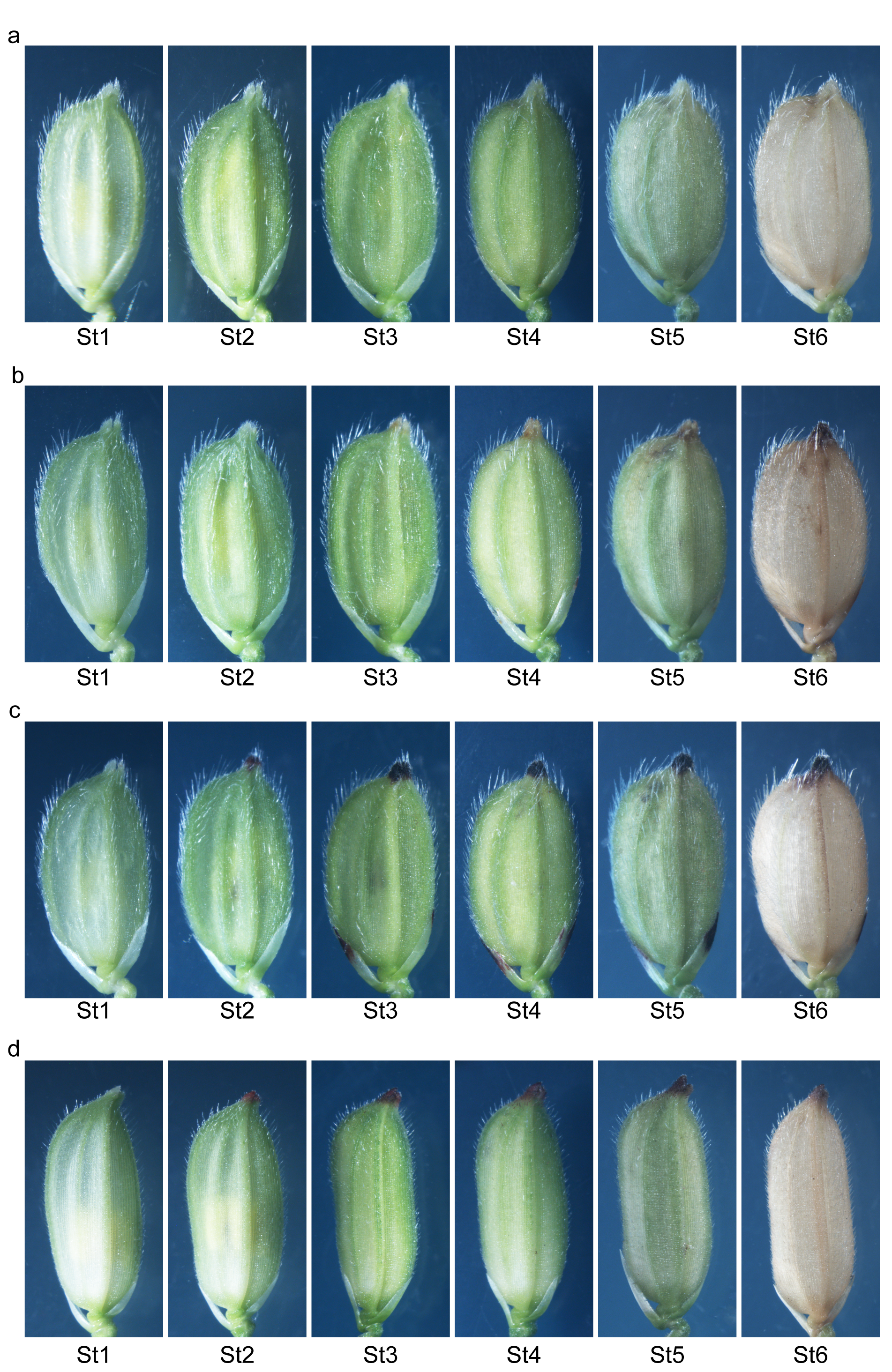

Supplement: Supplementary file 4 — Additional file 4: Fig. S4. Apiculus color development in Kitaake, XQZ and transgenic complemented lines. Apiculus color development of (a) Kitaake, (b) OsC1-transgenic complemented lines, (c) OsC1 and OsDFR-transgenic complemented lines, and (d) XQZ. St1 to St6 are − 1, 1, 7, 14, 21, and 26 days post heading. [file 12284_2021_480_MOESM4_ESM.tif]

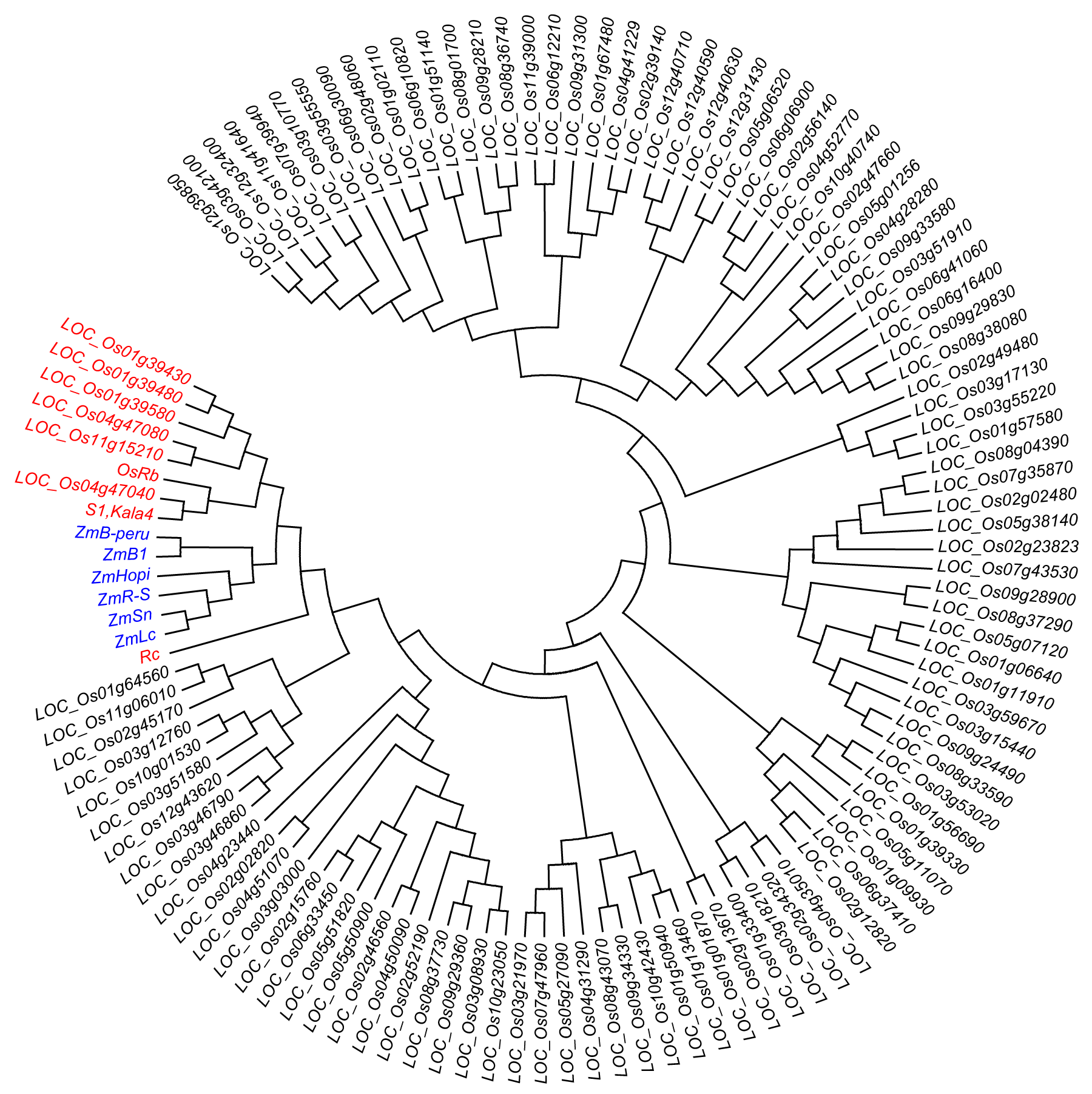

Supplement: Supplementary file 5 — Additional file 5: Fig. S5. Phylogenetic tree of all rice bHLH transcriptional factors (TFs) and known maize bHLH TFs associated with anthocyanin biosynthesis. The genes in blue denote known maize bHLH TFs associated with anthocyanin biosynthesis. The genes highlighted in red represented are the rice bHLH TFs closest to the known maize bHLH TFs. The tree was constructed using MEGA 5.2 and bootstrapped with 1000 replicates. [file 12284_2021_480_MOESM5_ESM.tif]

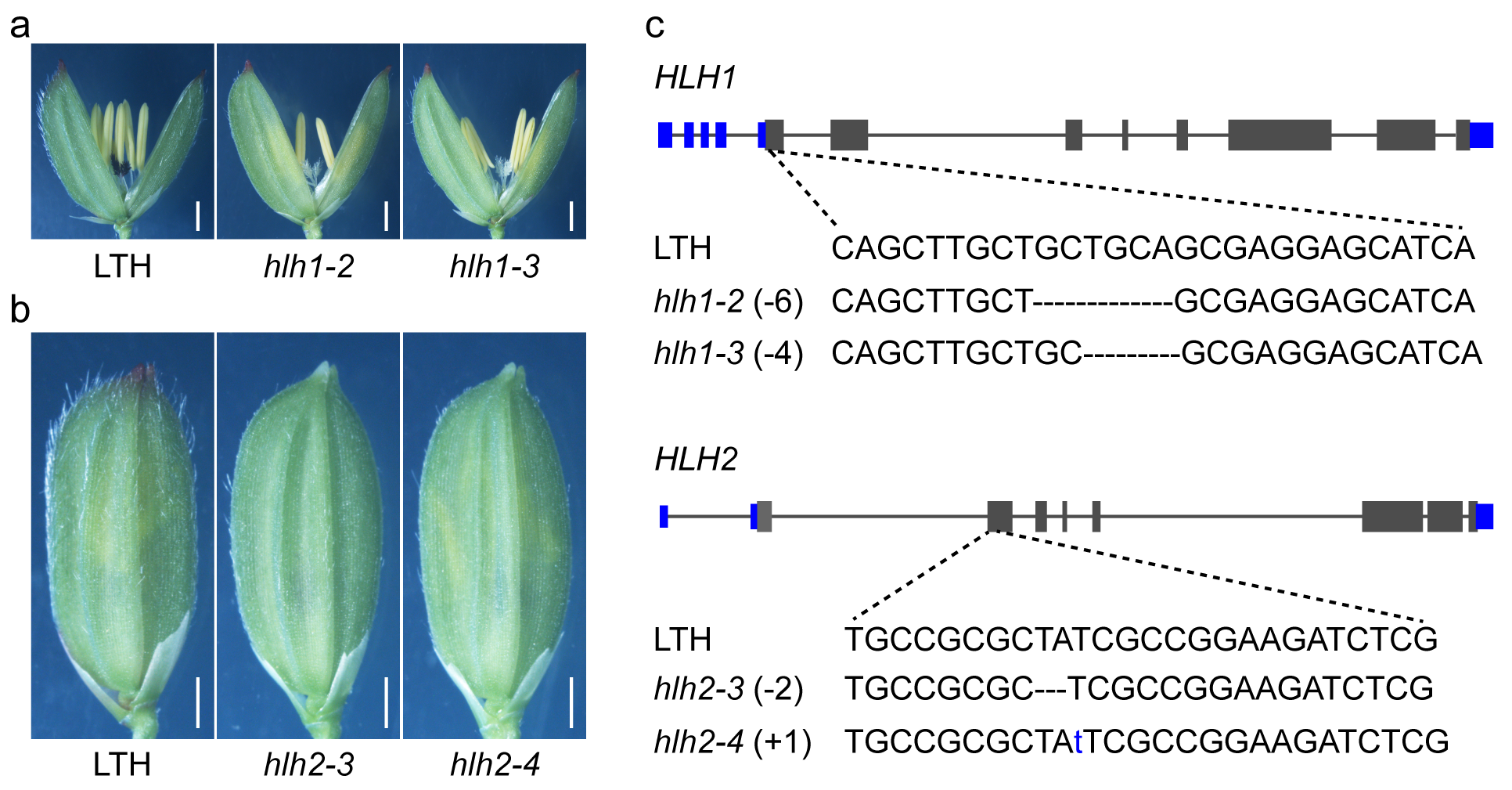

Supplement: Supplementary file 6 — Additional file 6: Fig. S6. Phenotypes of HLH1 and HLH2 knockout mutants in the LTH background. (a) the stigma color changed from purple to straw-white in hlh1 mutants. Bar, 1 mm. (b) Purple apiculus color is lost in hlh2 mutants. Bar, 1 mm. (c) Sequencing of the CRISPR/Cas9-targeted sites of HLH1- and HLH2- knockout lines. Plus (+) and minus (−) indicate base insertions (in blue) and deletions (by hyphen), respectively, relative to LTH. Gray boxes denote coding sequences of HLH1 and HLH2, and blue boxes are the untranslated regions. hlh1–2 and hlh1–3 are independent HLH1-transgenic knockout lines. hlh1–2 has a six-base deletion at position 52, causing a two-amino acids deletion. hlh1–3 has a four-base deletion at position 54, causing a premature termination of translation. hlh2–3 and hlh2–4 are independent HLH2-transgenic knockout lines. hlh2–3 has a two-base deletion, and hlh2–4 has a single base insertion, both causing premature termination of translation. [file 12284_2021_480_MOESM6_ESM.tif]

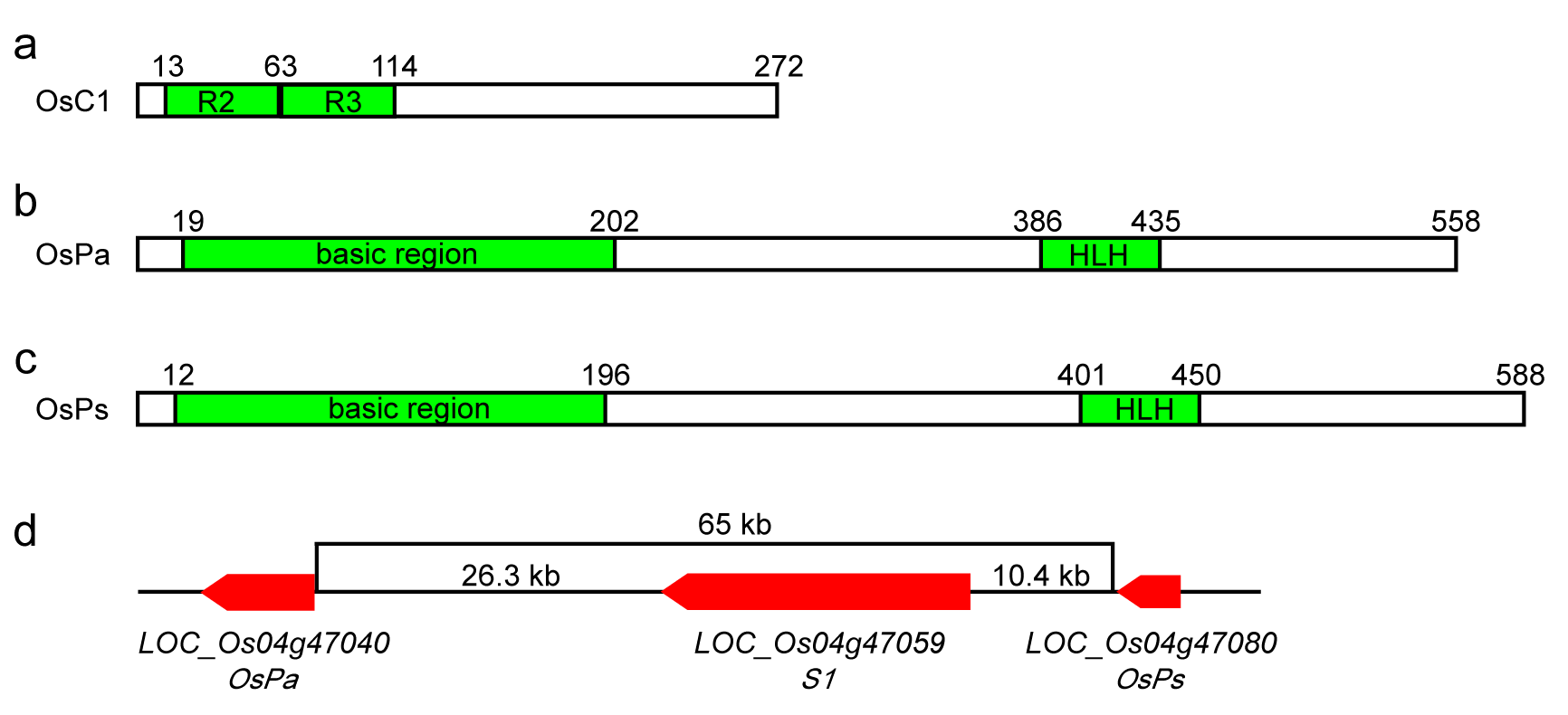

Supplement: Supplementary file 7 — Additional file 7: Fig. S7. Protein structures of OsC1, OsPa, OsPs and physical distance analysis of among three bHLH transcriptional factors (TFs). (a-c) Protein structures of OsC1, OsPa and OsPs. Numbers above the diagrams indicate residue positions. R2R3 repeats, basic regions and hydrophobic HLH regions are labelled with green boxes. (d) Physical distances separating three bHLH TFs on chromosome 4 indicated above the genes. [file 12284_2021_480_MOESM7_ESM.tif]

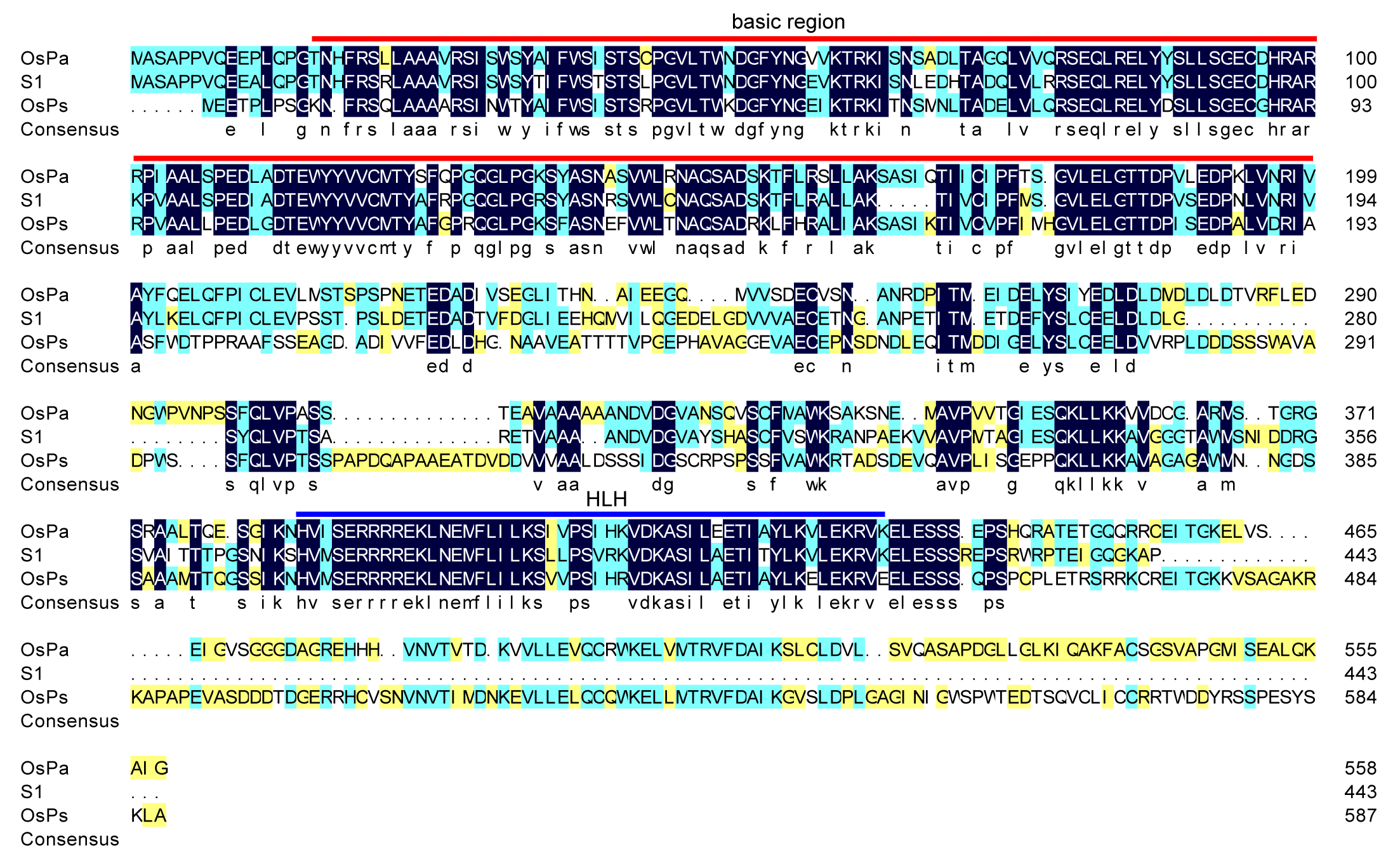

Supplement: Supplementary file 8 — Additional file 8: Fig. S8. Amino acid sequence alignments of OsPa, OsPs and S1 determined using DNAMAN. Consensus amino acids are shown at the bottom with lowercase letters; black indicates 100% identify; green indicates > 50% identify; and yellow indicates > 33% identify. Red and blue lines indicate the basic region and HLH domain, respectively. [file 12284_2021_480_MOESM8_ESM.tif]

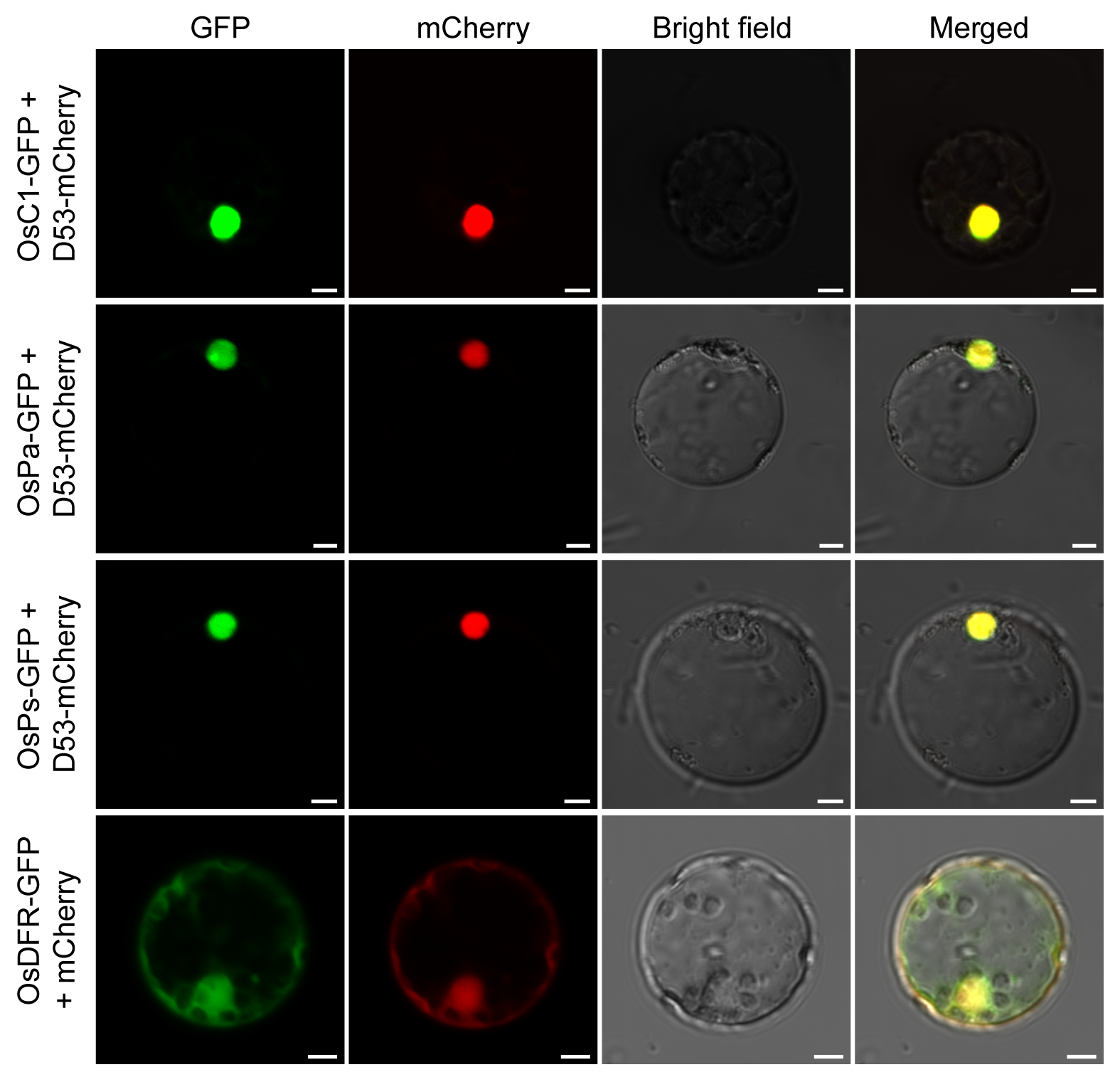

Supplement: Supplementary file 9 — Additional file 9: Fig. S9. Subcellular localization of OsC1, OsPa, OsPs and OsDFR in rice protoplasts. The OsC1-, OsPa-, OsPs-GFP fusion proteins were transiently co-expressed with nuclear marker D53-mCherry in rice protoplasts. OsDFR-GFP fusion protein was co-expressed with empty-mCherry in rice protoplasts. Left to right: images of GFP (green), mCherry (red), protoplast, and merged GFP and mCherry. [file 12284_2021_480_MOESM9_ESM.tif]

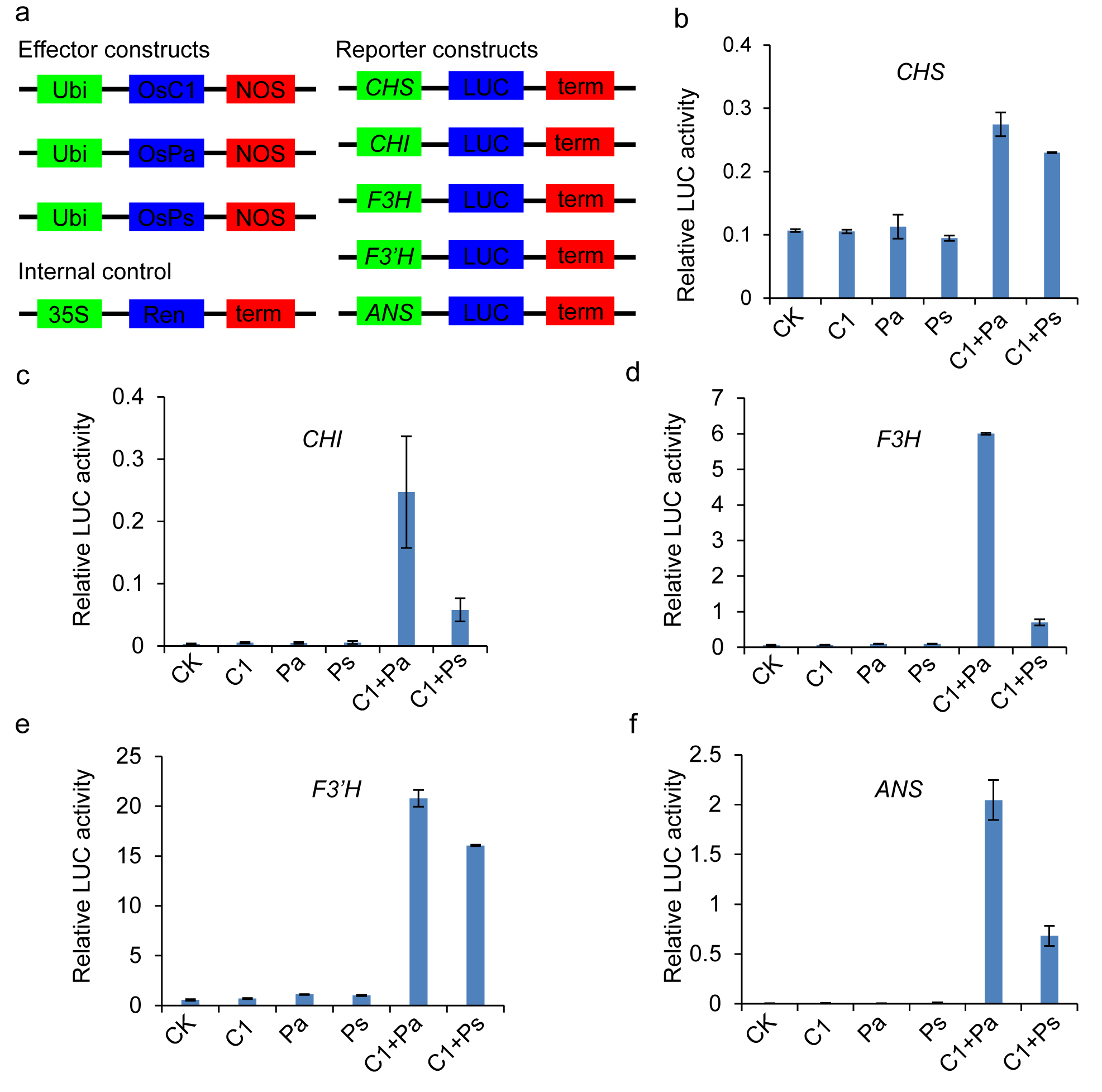

Supplement: Supplementary file 10 — Additional file 10: Fig. S10. Transient activation assays of OsC1, OsPa and OsPs on the promoters of five structural genes. (a) Schematic representation of the effector and reporter constructs. Full-length coding regions of OsC1, OsPa and OsPs under control of the ubiquitin promoter were used as the effectors. The Firefly luciferase gene LUC driven by the five structural genes promoters and the Renilla luciferase gene Ren driven by the 35S promoter were used as reporter and internal control, respectively. (b-f) Transient dual-luciferase assays were performed in Nicotiana benthamiana leaves to investigate the effects of OsC1, OsPa and OsPs on the transcriptional expression of structural genes. Relative LUC activity was measured by Firefly luciferase (LUC): Renilla luciferase (REN) ratio and data are presented as mean ± SD (n = 3). [file 12284_2021_480_MOESM10_ESM.tif]

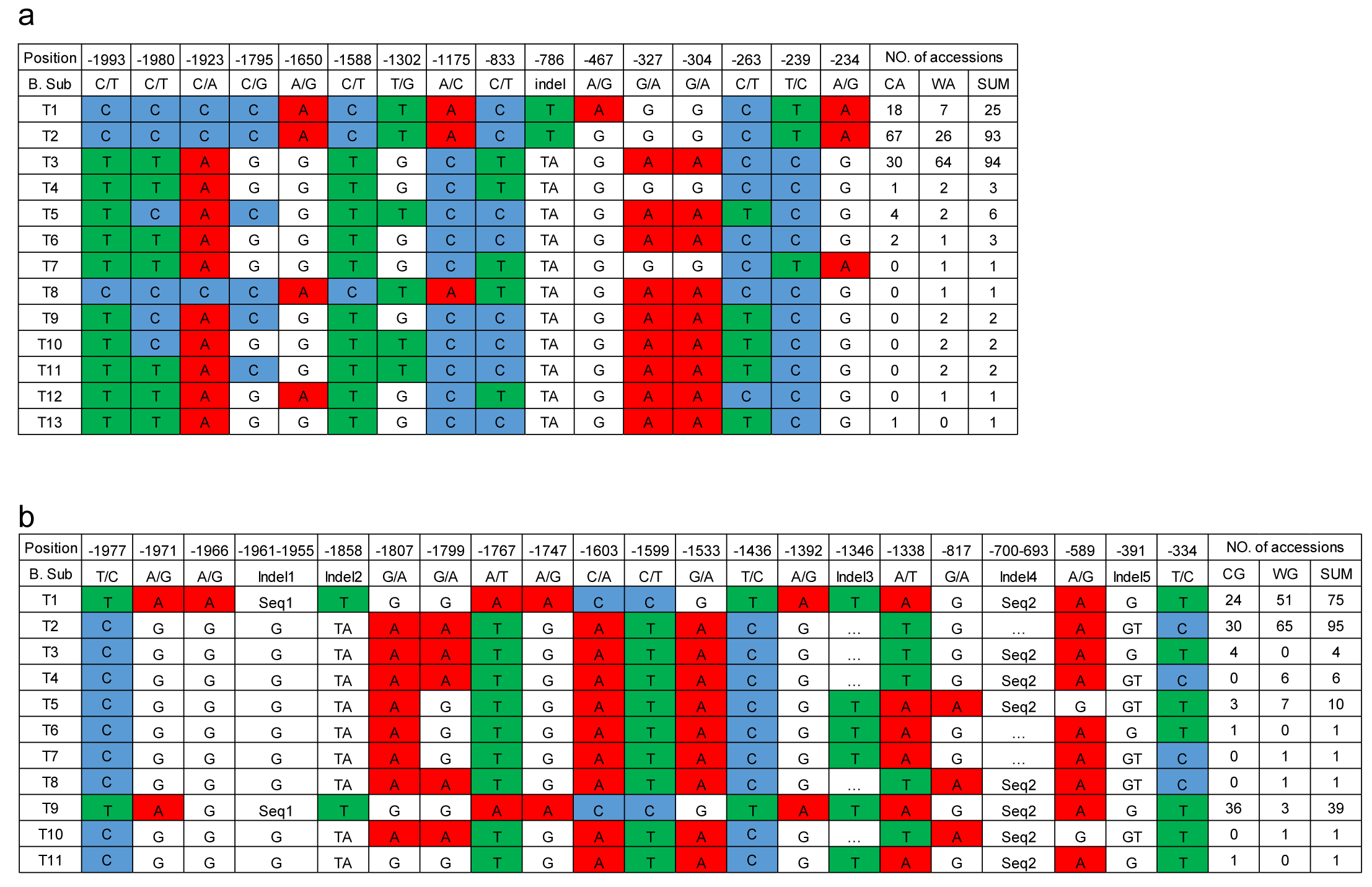

Supplement: Supplementary file 11 — Additional file 11: Fig. S11. Sequencing of 2.0 kb promoter regions of OsPa and OsPs in a panel of 234 rice accessions. Promoter sequence variations of OsPa (a) and OsPs (b) in the 234 rice accessions. B. sub are base substitutions. CA, colored apiculi; WA, straw-white apiculi; CG, colored stigmas; WG, straw-white stigmas. Deletion and insertion sites are indicated by dashed lines. Promoter sequence analysis of OsPa and OsPs was carried out with reference to the sequences of Nipponbare (T1). Seq1, CTAAAAT; Seq2, ACGACACT. The numbers at the top represented the base variation positions from the start codon. [file 12284_2021_480_MOESM11_ESM.tif]

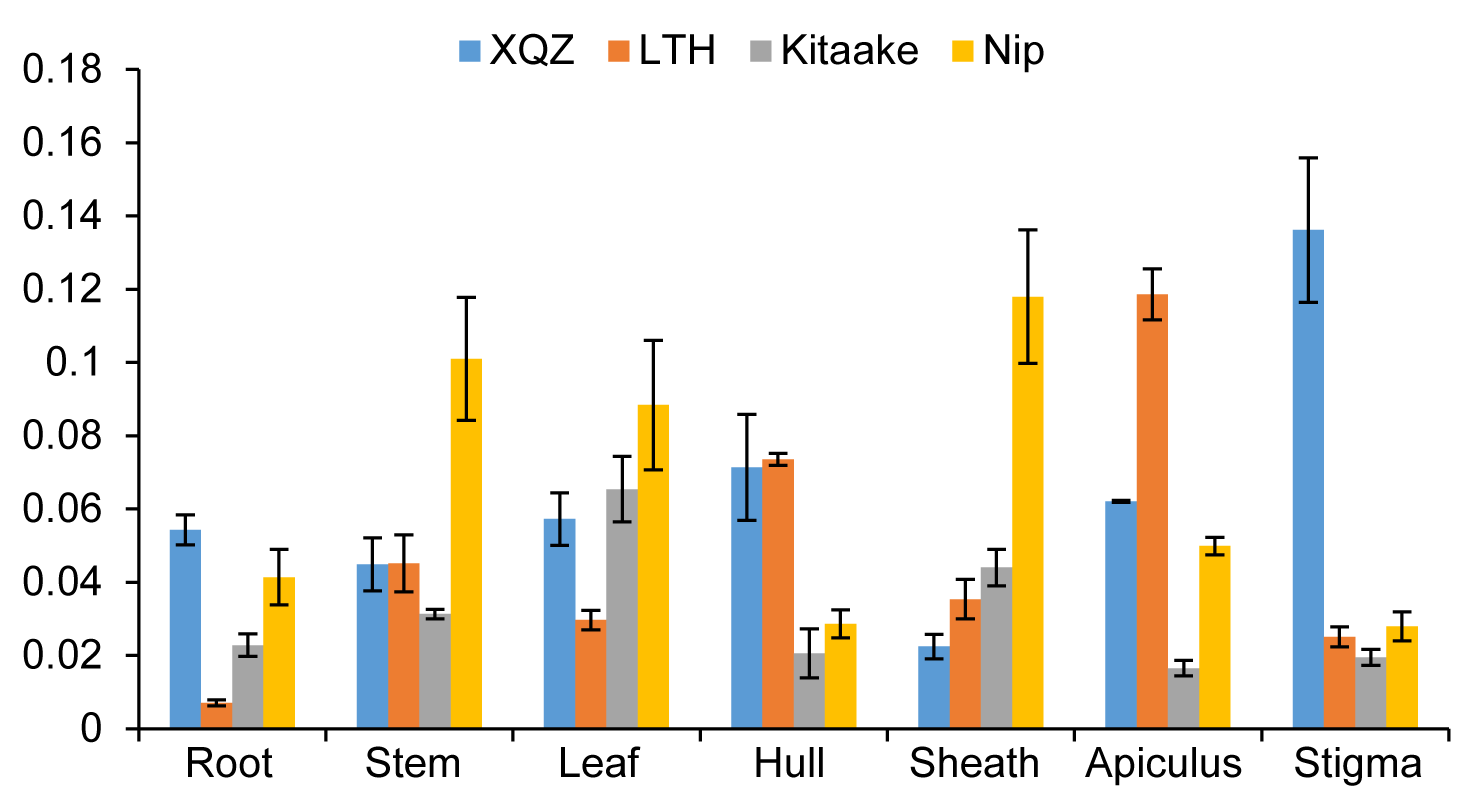

Supplement: Supplementary file 12 — Additional file 12: Fig. S12. Expression analysis of OsPAC1 in XQZ, LTH, Kitaake and Nipponbare (Nip). The samples were collected from roots of 10-day-old seedlings, stems, flag leaves, hulls, leaf sheaths, apiculi, and stigmas at heading. Data are presented as means ± SD (n = 3). [file 12284_2021_480_MOESM12_ESM.tif]
