# Supplementary material for: Determinant Factors and Regulatory Systems for Anthocyanin Biosynthesis in Rice Apiculi and Stigmas
Source: Rice (N Y). 2021 Apr 21;14:37. doi: 10.1186/s12284-021-00480-1 (PMC8060382; doi:10.1186/s12284-021-00480-1)
Supplement: Supplementary file 13 — Additional file 13: Table S1. Segregation of apiculi and stigma color in the F2 population derived from a cross between Kitaake and XQZ. [file 12284_2021_480_MOESM13_ESM.docx]

**Table S1.** Segregation of apiculi and stigma color in the F_2_ population derived from a cross between Kitaake and XQZ.

| Cross |  | No. of F_2_ individuals | | | | χ^2^_0.05_＜0.599 |
| --- | --- | --- | --- | --- | --- | --- |
|  |  | + + | +’− | - - | Total |  |
| XQZ ×Kitaake |  | 499 | 161 | 220 | 880 | 0.131 |

+ +: apiculi and stigmas both purple; +’ −: apiculi brown but stigmas straw-white; and − −: apiculi and stigmas both straw-white.
