# Supplementary material for: Determinant Factors and Regulatory Systems for Anthocyanin Biosynthesis in Rice Apiculi and Stigmas
Source: Rice (N Y). 2021 Apr 21;14:37. doi: 10.1186/s12284-021-00480-1 (PMC8060382; doi:10.1186/s12284-021-00480-1)
Supplement: Supplementary file 14 — Additional file 14: Table S2. Identification of tissue-specific bHLH-type genes in rice genome. [file 12284_2021_480_MOESM14_ESM.docx]

**Table S2.** Identification of tissue-specific bHLH-type genes in rice genome.

| MSU_Locus | Tentative name |
| --- | --- |
| *LOC_Os04g47080* | *HLH1* |
| *LOC*_*Os04g47040* | *HLH2* |
| *LOC*_*Os11g15210* | *HLH3* |
| *LOC*_*Os01g39580* | *HLH4* |
| *LOC*_*Os01g39480* | *HLH5* |
| *LOC_Os01g39430* | *HLH6* |
