# Supplementary material for: Determinant Factors and Regulatory Systems for Anthocyanin Biosynthesis in Rice Apiculi and Stigmas
Source: Rice (N Y). 2021 Apr 21;14:37. doi: 10.1186/s12284-021-00480-1 (PMC8060382; doi:10.1186/s12284-021-00480-1)
Supplement: Supplementary file 15 — Additional file 15: Table S3. Amino acids sequences alignment of three bHLH transcription factors. [file 12284_2021_480_MOESM15_ESM.docx]

**Table S3.** Amino acids sequences alignment of three bHLH transcription factors.

|  | Full length | |  | Basic region | |  | HLH domain | |
| --- | --- | --- | --- | --- | --- | --- | --- | --- |
|  | OsPa | S1 |  | OsPa | S1 |  | OsPa | S1 |
| S1 | 57.07% |  |  | 84.24% |  |  | 86.00% |  |
| OsPs | 51.00% | 43.81% |  | 75.68% | 73.51% |  | 88.00% | 84.00% |
