# Supplementary material for: Determinant Factors and Regulatory Systems for Anthocyanin Biosynthesis in Rice Apiculi and Stigmas
Source: Rice (N Y). 2021 Apr 21;14:37. doi: 10.1186/s12284-021-00480-1 (PMC8060382; doi:10.1186/s12284-021-00480-1)
Supplement: Supplementary file 20 — Additional file 20: Table S8. Analysis of haplotype combinations of the OsC1, OsDFR, OsPa and OsPs genes in lines with apiculus and stigma colors. [file 12284_2021_480_MOESM20_ESM.docx]

**Table S8.** Analysis of haplotype combinations of the *OsC1*, *OsDFR*, *OsPa* and *OsPs* genes in lines with apiculus and stigma colors.

| Genotype group | *OsC1* | *OsDFR* | *OsPa* | *OsPs* | Apiculus  color | Stigma  color | No. of *indica*  accessions | No. of *japonica* accessions | No. of total accessions | Frequency of genotype (%) |
| --- | --- | --- | --- | --- | --- | --- | --- | --- | --- | --- |
| I | +* | + | + | + | purple | purple | 23 | 18 | 41 | 23.30 |
| II | + | + | + | - | purple | straw-white | 2 | 0 | 2 | 1.14 |
| III | + | - | + | + | brown/red | straw-white | 4 | 18 | 22 | 12.50 |
| IV | - | + | + | + | straw-white | straw-white | 65 | 29 | 94 | 53.41 |
| V | - | - | + | + | straw-white | straw-white | 3 | 12 | 15 | 8.52 |
| VI | - | + | + | - | straw-white | straw-white | 2 | 0 | 2 | 1.14 |
| Total |  |  |  |  |  |  | 99 | 77 | 176 | 100 |

*: +, functional allele of *OsC1*, *OsDFR*, *OsPa* or *OsPs*; -, non-functional allele of *OsC1*, *OsDFR*, *OsPa* or *OsPs*
